# RECQL4 affects MHC class II‐mediated signalling and favours an immune‐evasive signature that limits response to immune checkpoint inhibitor therapy in patients with malignant melanoma

**DOI:** 10.1002/ctm2.70094

**Published:** 2025-01-15

**Authors:** Sara Egea‐Rodriguez, Renáta Váraljai, Thierry M. Nordmann, Restuan Lubis, Manuel Philip, Florian Rambow, Alexander Roesch, Michael Flaig, Susanne Horn, Raphael Stoll, Fang Zhao, Annette Paschen, Bert Klebl, Ian D. Hickson, Dirk Schadendorf, Matthias Mann, Iris Helfrich

**Affiliations:** ^1^ Department of Dermatology and Allergy University Hospital of Munich Ludwig‐Maximilian‐University Munich Germany; ^2^ German Cancer Consortium (DKTK) Partner Site Munich Munich Germany; ^3^ Skin Cancer Unit of the Dermatology Department Medical Faculty West German Cancer Center University Duisburg‐Essen Essen Germany; ^4^ German Cancer Consortium (DKTK) Partner Site Essen/Düsseldorf Essen Germany; ^5^ Proteomics and Signal Transduction Max Planck Institute of Biochemistry Martinsried Germany; ^6^ Lead Discovery Center GmbH Dortmund Germany; ^7^ Department of Applied Computational Cancer Research Institute for AI in Medicine (IKIM) University Hospital Essen University Duisburg‐Essen Essen Germany; ^8^ Rudolf Schönheimer Institute of Biochemistry Medical Faculty of the University of Leipzig Leipzig Germany; ^9^ Biomolecular Spectroscopy and RUBiospec NMR, Faculty of Chemistry and Biochemistry Ruhr University of Bochum Bochum Germany; ^10^ Center for Chromosome Stability Department of Cellular and Molecular Medicine University of Copenhagen Copenhagen N Denmark; ^11^ Present address: Research Center for Environmental Health Helmholtz Center Munich Ingolstädter Landstraße 1 Neuherberg 85764 Germany

**Keywords:** immune evasion, immunotherapy, melanoma, MHC class II, RECQL4

## Abstract

**Background:**

Cancer immunotherapy has transformed metastatic cancer treatment, yet challenges persist regarding therapeutic efficacy. RECQL4, a RecQ‐like helicase, plays a central role in DNA replication and repair as part of the DNA damage response, a pathway implicated in enhancing efficacy of immune checkpoint inhibitor (ICI) therapies. However, its role in patient response to ICI remains unclear.

**Methods:**

We analysed whole exome and bulk RNA sequencing data from a pan‐cancer cohort of 25 775 patients and cutaneous melanoma cohorts (untreated: *n* = 471, anti‐progressive disease [PD]‐1 treated: *n* = 212). *RECQL4* copy number variations and expression levels were assessed for patient outcomes. We performed gene set enrichment analysis to identify RECQL4‐dependent signalling pathways and explored the association between *RECQL4* levels and immunoscores. We evaluated the interplay of ICI response and *RECQL4* expression in melanoma cohorts of 95 responders and 85 non‐responders prior to and after ICI‐targeted therapy and tested the prognostic power of *RECQL4*. Finally, we generated genetically engineered RECQL4 variants and conducted comprehensive multi‐omic profiling, employing techniques such as liquid chromatography with tandem mass spectrometry, to elucidate mechanistic insights.

**Results:**

We identified RECQL4 as a critical negative regulator of poor prognosis and response to ICI therapy, but also demonstrated its suitability as an independent biomarker in melanoma. High tumour purity and limited signatures of tumour immunogenicity associated with response to anti‐PD‐1 correlated with high RECQL4 activity. We found alterations in the secretion profile of immune regulatory factors and immune‐related pathways robustly suppressed in tumours with high *RECQL4* levels, underscoring its crucial role in fostering immune evasion. Mechanistically, we identified RECQL4‐mediated regulation of major histocompatibility complex class II molecule expression and uncovered class II major histocompatibility complex transactivator as a mediator bridging this regulation.

**Conclusions:**

Our findings unraveled the pivotal role of RECQL4 in immune modulation and its potential as both a predictive biomarker and therapeutic target for optimising immunotherapeutic strategies across various cancer types.

**Highlights:**

- High RECQL4 expression limits survival and can act as an independent prognostic factor in melanoma patients.
- RECQL4 has the potential to act as a negative feedback mediator of immune checkpoint‐targeted therapy by limiting signatures associated with therapeutic efficacy.
- RECQL4 favours an immune‐evasive phenotype by downregulating major histocompatibility complex class II molecules.

## Graphical abstract

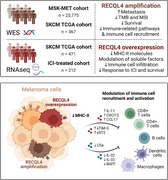

## 1 BACKGROUND

The DNA damage response (DDR) is a double‐edged sword since it is responsible for maintaining genome stability in normal cells, but also utilised by cancer cells to overcome therapy‐induced DNA damage and cell death. Deficiency in DNA repair mechanisms is associated with increased genomic instability and cancer development and has been discussed recently for its therapeutic potential in the context of immune checkpoint inhibitor (ICI)‐based therapy.^1^ ICIs are monoclonal antibodies that target surface proteins such as progressive disease (PD)‐1 and CTLA‐4, thereby restoring T‐cell‐mediated anti‐tumour activity. ICIs have become the gold standard for advanced cancer treatment, offering patients with metastatic disease a significant improvement in long‐term survival prospects. They have also substantially improved the treatment outcome, with an increase in progression‐free survival (PFS) observed in individuals at the early stages of the cancer disease, including patients suffering from malignant melanoma.^2^ Despite impressive clinical results observed, the majority of patients either experience treatment failure or develop resistance to immunotherapy.^3^ Interestingly, patients with deficiency in DNA mismatch repair (dMMR) or a high level of microsatellite instability (MSI) show enhanced efficacy to ICI‐based therapy.^4^ These observations highlight the potential of targeting the DDR to improve current treatment strategies for cancer patients. This notion is further reinforced by the concept of synthetic lethality^5^ and that DDR‐targeting drugs, such as poly (ADP‐ribose) polymerase inhibitors, are already successfully used in the clinic.^6^, ^7^

Helicases are important genome caretakers and part of the DDR cascade. They utilise the energy derived from the hydrolysis of adenosine triphosphate (ATP) to unwind double‐stranded nucleic acids, thereby facilitating essential processes such as replication, recombination, transcription and repair.^8^ Numerous genetic diseases characterised by a predisposition to cancer are linked to mutations in genes encoding DNA helicases.^9^, ^10^ Loss of function mutations in RecQ like helicase 4 (RECQL4) are linked to different autosomal recessive and cancer‐prone syndromes such as Rothmund–Thomson syndrome, Baller–Gerold syndrome and RAPADILINO. These patients typically develop osteosarcomas and lymphomas.^11^ In addition, the region of chromosome 8q where *RECQL4* is located is frequently amplified in various tumours^12^, ^13^; a region that is close to the *MYC* oncogene locus. Moreover, previous research has indicated a correlation between elevated RECQL4 levels, tumour aggressiveness and poor prognosis in a subset of cancer types, comprising prostate,^14^ breast,^15^ ovarian,^16^ gastric,^17^, ^18^ oesophageal cancer^19^, colon adenocarcinoma,^20^ hepatocellular carcinoma^21^ and glioma.^22^

Continuous upregulation of RECQL4 in highly proliferative conditions, such as cancer development, argues for its suitability as a therapeutic target. Yet, the precise role of RECQL4 in cancer progression requires further elucidation and overarching cancer studies are lacking. Moreover, the mode‐of‐action of RECQL4‐dependent signatures on signalling pathways that alter the efficacy of ICI‐based therapy is poorly understood.

In this study, we have uncovered RECQL4 as a critical negative regulator of ICI therapy, dampening immune cell signatures crucial for an effective ICI response. Notably, increased RECQL4 activity correlates with diminished expression of major histocompatibility complex class II (MHC‐II) molecules and shifts in the secretion profile of immune regulatory factors, underscoring its pivotal role in fostering immune evasion. Importantly, these findings position RECQL4 as a promising therapeutic target. Our results emphasise the specificity of RECQL4‐mediated MHC‐II downregulation, thereby implicating an as‐yet‐unknown distinct mechanism.

## 2 METHODS

### 2.1 Patient cohorts and databases

We followed the SAGER guidelines regarding sex and gender equity in research.^23^ For copy number analysis, we investigated the Memorial Sloan Kettering—Metastatic Events and Tropisms (MSK‐MET) cohort^24^ including 25 775 patients spanning 50 cancer types; and the Skin Cutaneous Melanoma (SKCM) cohort from The Cancer Genome Atlas (TCGA, Firehose Legacy, RRID:SCR_003193). In these cohorts, *RECQL4* copy number alterations (CNA) and available clinical data were analysed. These data were taken from the cBioPortal for cancer genomics online database^25^, ^26^ (https://www.cbioportal.org/, RRID:SCR_014555). For transcript level analysis, we explored the SKCM TCGA Firehose Legacy cohort and four independent cohorts consisting of ICI‐treated melanoma patients as formerly published in Liu et al.,^27^ Hugo et al.,^28^ Gide et al.^29^ and Riaz et al.^30^ From these cohorts, we selected only the samples from pre‐treatment biopsies with cutaneous melanoma subtype. We extracted the clinico‐pathological and *RECQL4*, *MYC* and *MKI67* expression in transcripts per million (TPM) mapped reads data from the PhenoTImE webtool^31^ (https://doc.hornlab.org/shiny/cru337phenotime/). RNA sequencing (RNA‐seq) expression data from TCGA (RNASeqV2) was processed and normalised using RNA‐seq by Expectation‐Maximisation to generate TPM. Table 1 shows a summary of the analysed cohorts for *RECQL4* CNA and gene expression levels.

**TABLE 1:** Clinical data from the patient cohorts.

Genomic alterations cohorts		
	MSK‐MET	SKCM TCGA
Sex, *N* (%)		
Female	13 485 (52.3%)	138 (37.6%)
Male	12 286 (47.65%)	229 (62.4%)
NA	4 (.015%)	0
Age, median (min‒max)	61 (12‒90)	56 (15‒87)
Sample type, *N* (%)		
Primary	15 632 (60.6%)	0
Metastasis	10 143 (39.4%)	367 (100%)

RNA‐seq cohorts		
	ICI treated	SKCM TCGA
Sex, *N* (%)		
Female	85 (40.1%)	179 (38.0%)
Male	127 (59.9%)	292 (62.0%)
Age, median (min‒max)	59 (19‒90)	58 (15‒90)
Sample type, *N* (%)		
Primary	0	103 (21.9%)
Metastasis	212 (100%)	368 (78.1%)

*Note*: Numbers (*N*) and percentages (in brackets) of patients included in the genomic alterations and RNA‐seq cohorts.

Abbreviations: ICI, immune checkpoint inhibitor; MSK‐MET, Memorial Sloan Kettering—Metastatic Events and Tropisms; NA, non‐available information; RNA‐seq, RNA sequencing; SKCM TCGA, Skin Cutaneous Melanoma cohort from The Cancer Genome Atlas.

### 2.2 *RECQL4* copy number and gene expression analysis

*RECQL4* CNA data from the cBioPortal classified the copy number levels using the Genomic Identification of Significant Targets in Cancer version 2.0 algorithm (GISTIC2.0, RRID:SCR_000151).^32^ According to the copy number, the samples were classified in four levels: (1) loss of heterozygosity (LOH), which indicates a shallow loss, probably a heterozygous deletion; (2) diploidy; (3) gain, which implies a low‐level gain of limited additional copies and generally of a broad segment; and (4) high‐level amplification (HIGH AMP), which indicates a gain of several additional copies and is generally focal. We mainly focused on the differences between the diploid and high amplification levels for the *RECQL4* locus. Regarding transcript levels, we divided the samples in ‘low‐’ and ‘high‐expression groups’, individually for each cohort, by *k*‐means clustering using R language. We then merged the ICI‐treated cohorts and used these groups for the comparison of different clinical parameters and performance of survival analyses. In Table 2, details about the individual ICI‐treated cohorts are presented.

**TABLE 2:** Clinical data from the immune checkpoint inhibitor (ICI)‐treated melanoma cohort.

	Liu et al.	Hugo et al.	Gide et al.	Riaz et al.	Merged
Sex, *N* (%)					
Female	36 (40.9%)	8 (30.8%)	25 (34.7%)	16 (61.5%)	85 (40.1%)
Male	52 (59.1%)	18 (69.2%)	47 (65.3%)	10 (38.5%)	127 (59.9%)
Age, median (min‒max)	NA	60.5 (19‒84)	62 (24‒90)	52 (41‒81)	59 (19‒90)
Sample type, *N* (%)					
Primary	0	0	0	0	0
Metastasis	88 (100%)	26 (100%)	72 (100%)	26 (100%)	212 (100%)
Disease status, *N* (%)					
IIIC	9 (10.2%)	0	0	0	9 (4.2%)
M1a	5 (5.7%)	1 (3.8%)	0	6 (23.1%)	12 (5.7%)
M1b	11 (12.5%)	3 (11.5%)	0	4 (15.4%)	18 (8.5%)
M1c	63 (71.6%)	21 (80.8%)	0	12 (46.1%)	96 (45.3%)
NA	0	1 (3.8%)	72 (100%)	4 (15.4%)	77 (36.3%)
Mutational subtype, *N* (%)					
BRAF	35 (39.8%)	13 (50.0%)	25 (34.7%)	9 (34.6%)	82 (38.7%)
NF1	6 (6.8%)	2 (7.7%)	0	1 (3.8%)	9 (4.2%)
RAS	22 (25.0%)	5 (19.2%)	0	6 (23.1%)	33 (15.6%)
Triple Wt	25 (28.4%)	6 (23.1%)	0	8 (30.8%)	39 (18.4%)
NA	0	0	47 (65.3%)	2 (7.7%)	49 (23.1%)
Treatment, *N* (%)					
Nivolumab	34 (38.6%)	0	16 (22.2%)	26 (100%)	76 (35.8%)
Pembrolizumab	54 (61.4%)	26 (100%)	56 (77.8%)	0	136 (64.2%)
Prior ipilimumab, *N* (%)					
CTLA4‐naive	55 (62.5%)	26 (100%)	41 (56.9%)	15 (57.7%)	137 (64.6%)
CTLA4‐experienced	33 (37.5%)	0	31 (43.1%)	11 (42.3%)	75 (35.4%)
RECISTv1.1, *N* (%)					
CR/PR	36 (40.9%)	13 (50.0%)	39 (54.2%)	7 (26.9%)	95 (44.8%)
SD/MR	15 (17.0%)	0	11 (15.3%)	6 (23.1%)	32 (15.1%)
PD	37 (42.1%)	13 (50.0%)	22 (30.5%)	13 (50.0%)	85 (40.1%)

*Note*: Numbers (*N*) and percentages (in brackets) of patients included in the ICI‐treated melanoma cohort. Response Evaluation Criteria In Solid Tumours version 1.1 (RECISTv1.1) indicates the classification of patients’ response in CR, PR, SD, MR and PD according to the published guidelines.^33^

Abbreviations: CR, complete response; MR, mixed response; NA, non‐available information; PD, progressive disease; PR, partial response; SD, stable disease.

### 2.3 Survival analysis

We used the ‘survival’ R package (RRID:SCR_021137) to conduct Kaplan–Meier analyses. We compared the disease‐free survival (DFS), PFS and/or overall survival (OS) fraction between the different *RECQL4* copy number/expression groups in several cohorts using the log rank test. We utilised the ‘survminer’ (RRID:SCR_021094) and ‘ggplot2’ (RRID:SCR_014601) R packages to estimate and visualise survival curves. To assess whether *RECQL4* functions as an independent prognostic factor, we performed a multivariate Cox proportional hazard (CoxPH) model analysis where we simultaneously evaluated the effect of clinical and genomic variables on survival time in the ICI‐treated cohort. We included clinical variables based on previously demonstrated association with survival and on availability among the cohorts. We also included *KI67* and *MYC* low‐ and high‐expression groups next to *RECQL4*. We used Wald test, Likelihood ratio test and score (log rank) test to evaluate the *p*‐values of variables. Results were represented as forest plots showing the hazard ratio (HR) and 95% confidence intervals by using the ‘forestplot’ (RRID:SCR_023633) R package.

### 2.4 Genomic analysis

We obtained the genomic data of the MSK‐MET cohort through cBioPortal. Fraction of genome altered (FGA) was defined as the fraction of the genome with absolute log2 copy ratios larger than .2, as previously described.^34^ MSI type was categorised by the MSIsensor score (RRID:SCR_006418) according to Niu et al.^35^ We calculated the tumour mutational burden (TMB) by dividing the total number of non‐synonymous mutations by the number of bases sequenced for each sample. We considered the samples with ≥10 mutations/megabase (mut/Mb) as high TMB.

### 2.5 Gene set enrichment analysis

For gene set enrichment analysis (GSEA), we first derived the differentially expressed genes in diploid versus high‐amplified *RECQL4* samples within the SKCM TCGA cohort. Next, we ranked the genes according to the logarithm of the fold change (FC) and the *p*‐value of the differential expression and performed a GSEA using the Hallmark Gene Set of the Molecular Signatures Database (MSigDB, RRID:SCR_022870).^36^ GSEA was executed using ‘fgsea’ R package (RRID:SCR_020938). Normalised enrichment score (NES), *p*‐value and the false discovery rate (FDR) adjusted *p*‐value corrected by Benjamini–Hochberg multiple test (*q*‐value) were calculated. In addition, we analysed enrichment in gene ontology (GO) terms defining biological processes using ShinyGO 0.76.2 webtool (RRID:SCR_019213).^37^ The same procedures were repeated with Liu et al.^27^ cohort as a validation of the results.

### 2.6 Immune infiltration analysis

We employed the ‘Immune‐sCNA’ module of Tumour Immune Estimation Resource version 2 (TIMER2.0)^38^ webtool (http://timer.cistrome.org/, RRID:SCR_018737) to compare immune infiltration distribution according to *RECQL4* copy number in SKCM samples from the TCGA database. Intra‐tumoural infiltration by the different immune cell subtypes was estimated by the webtool according to xCell pipeline.^39^ In addition, we explored the correlation between *RECQL4* mRNA expression and immune cell infiltrate using the ‘Immune‐Gene’ module of TIMER 2.0. Partial Spearman's correlation adjusted by tumour purity was calculated. We also compared the total immune score between the low and high *RECQL4* expression groups from SKCM TCGA cohort. To evaluate if the observed differences in immune infiltration are related to anti‐PD‐1 response, we compared the tumour immunogenicity associated with response to anti‐PD‐1 (TIARA‐PD‐1) between low and high *RECQL4* expression groups in the ICI‐treated cohort. TIARA‐PD‐1 was established by Campbell et al.^40^ based on RNA‐seq data to compile gene expression signatures of immune cells linked to anti‐PD‐1 response into a single metric.

### 2.7 Physiological and pathophysiological expression of *RECQL4*

To examine the differential mRNA expression of *RECQL4* between tumour and healthy tissues across all TCGA cancer types, we employed ‘Gene_DE’ module of TIMER2.0. The log_2_ TPM transformed expression data are presented in box plots and the statistical significance was computed by the Wilcoxon rank sum test. Because the TCGA dataset only contains tumour and metastatic tissue for SKCM, we used Gene Expression Profiling Interactive Analysis version 2 (GEPIA2) webtool^41^ (http://gepia.cancer‐pku.cn/index.html, RRID:SCR_018294) to compare tumour TCGA data and matched data from corresponding healthy tissue from the Genotype‐Tissue Expression (GTEx, RRID:SCR_013042) dataset.

### 2.8 Generation of RECQL4‐overexpressing cell lines

A375 (#CRL‐1619, ATCC; RRID:CVCL_0132) cells were cultured in RPMI 1640 medium (#21875‐034; Gibco, Thermo Fisher Scientific) supplemented with 10% foetal bovine serum (FBS, #A15‐101; PAA Laboratories), 2 mM L‐glutamine (#25030‐024; Gibco, Thermo Fisher Scientific) and 100 IU/mL penicillin and streptomycin (Pen Strep, #15140‐122; Gibco, Thermo Fisher Scientific). No further reauthentication of the aforementioned cell line was conducted for the purposes of this study beyond the authentication procedures undertaken by the providers. All cell lines were cultured at 37°C with 5% CO_2_ and subjected to routine testing for mycoplasma contamination using the Mycoplasma PCR Detection Kit (#G238; Applied Biological Materials Inc.).

For RECQL4 overexpression, A375 cells were transfected with pcDNA3.1(+) vector containing RECQL4 or the helicase‐dead RECQL4^K508A^ mutant, harbouring a point mutation of the conserved lysine in the Walker A motif of the SFII helicase domain, both with a Flag and 6xHis at the N‐terminus. We also generated the empty vector control in A375. The vectors were synthesised by GenScript Biotech. JetPRIME transfection reagent (#101000015; Polyplus, Sartorius) was used for this purpose following the protocol provided by the company. Both control non‐transfected and transfected cells were treated with geneticin disulphate (G418, #2039.2; Carl Roth) at a concentration of .5 and 1 mg/mL for selection of polyclonal stable colonies during at least 1 week until the non‐transfected cells were dead. Protein expression of RECQL4 after transfection was measured routinely by Western blot.

### 2.9 Protein isolation and Western blot

For protein isolation, A375 transfected cells were washed twice in cold DPBS and lysed in ice‐cold Cell Lysis Buffer (Cell Signalling Technology Cat# 9803) supplemented with .1% SDS, 1 mM PMSF and cOmplete Mini EDTA‐free Protease Inhibitor Cocktail (Roche Cat# 11836170001) to ensure protein stability. Lysates were cleared by centrifugation at 4°C and protein concentrations were determined using the Pierce BCA Protein Assay Kit (Thermo Fisher Scientific Cat# 23227). Equivalent quantities of protein were loaded and separated on an 8% SDS‐PAGE gel, and then transferred to polyvinylidenfluorid membranes by using the Trans‐Blot Turbo Transfer System (Bio‐Rad). The membranes were blocked with 5% milk in Tris‐buffered saline‒.1% Tween 20 (TBST) for 30 min at RT, washed in TBST (3 × 10 min) and incubated overnight at 4°C with the following primary antibodies: anti‐actin (1:10 000; Thermo Fisher Scientific Cat# ICN691001 [also 08691001], RRID:AB_2335127) and anti‐RECQL4 (1:1000; Thermo Fisher Scientific Cat# PA5‐75608, RRID:AB_2719336). The membranes were then washed in TBST (3 × 10 min) and incubated for 1 h at RT with the following secondary antibodies: anti‐mouse immunoglobulin G (IgG) HRP (1:5000; SouthernBiotech Cat# 1031‐05, RRID:AB_2794307) and anti‐rabbit IgG HRP (1:5000; SouthernBiotech Cat# 4030‐05, RRID:AB_2687483). All antibodies were diluted in 5% milk in TBST. After washing in TBST, chemiluminescence was detected with the Sapphire Biomolecular Imager (Azure Biosystems) using Clarity Western ECL Substrate (Bio‐Rad Cat# 170‐5060). Immunoblot band intensities were quantified using Image Lab Software 6.1 (Bio‐Rad, RRID:SCR_014210).

### 2.10 Measurement of RECQL4 HRR activity

To quantify the differences in RECQL4 activity among A375 pcDNA3.1, A375 pcDNA3.1 RECQL4^K508A^ and A375 pcDNA3.1 RECQL4, the transfected cells were resuspended in free‐phenol red RPMI supplemented with 10% FBS, .5 mg/mL G418 and 2 mM glutamine. Cells were then co‐transfected with homologous recombination repair (HRR) reporter and endonuclease plasmids using TransIT‐LT1 transfection reagent (Mirus Cat# MIR2300). At 48 h post‐transfection, the recovery of intact luciferase was detected by using Nano‐Glo Luciferase Assay System (Promega, Cat# N1120) to calculate the HRR level. Corresponding volumes of the detection reagent and culture medium were added to each well. Finally, the assay plate was read after 10 min using the EnVision multimode plate reader (PerkinElmer).

### 2.11 Liquid chromatography with tandem mass spectrometry

#### 2.11.1 Sample preparation for mass spectrometry

A375 transfected cells were plated and harvested in parallel per condition in quadruplicates. At a confluence of 80%, cells (1 × 10^6^) were trypsinised, centrifuged (1000 rpm; 5 min) and washed two times with cold DPBS. Following further centrifugation (maximum speed, 5 min, 4°C), cells were pelleted and preserved in liquid nitrogen. Pellets were then resuspended in lysis buffer (10% acetonitrile [ACN], 60 mM triethylammonium bicarbonate, pH 8.5, 5 mM tris(2‐carboxyethyl)phosphine, 25 mM chloroacetamide), followed by heating (76°C for 10 min). Samples were then sonicated in the Bioruptur (15 cycles, 30 s each) and heated again (76°C, 30 min). We determined the proteins concentration using the tryptophan assay. LysC/Trypsin (1:100) was added to the samples, incubated for 16 h (37°C, 750 rpm) and quenched with TFA (1% final concentration).

### 2.12 Loading of peptide samples on Evotips

Peptides were measured by tryptophan assay and 200 ng of each sample was loaded on Evotip Pure (Evosep). For this, tips were activated with 1‐propanolol for 3 min, washed with 50 µL 100% ACN/.1% FA, re‐activated with 1‐propanolol for 3 min, equilibrated with 50 µL .1% FA in mass spectrometry (MS)‐grade H_2_O, followed by sample loading directly into 70 µL .1% FA in MS‐grade H_2_O pipetted into each tip. Tips were then washed with 50 µL .1% FA in MS‐grade H_2_O twice, filled up with 100 µL of .1% FA in MS‐grade H_2_O and centrifuged briefly. Centrifugation was performed at 500 g for 1 min, unless otherwise stated.

### 2.13 Liquid chromatography

Peptides were separated by hydrophobicity and introduced into the mass spectrometer with the EvoSep One liquid chromatography system with a standard 21 min (60 SPD) gradient. We used an 8 cm column with 150 µm inner diameter and 1.5 µm C18 beads (PepSep) heated to 50°C and coupled to an ID 30 µm stainless steel electrospray emitter (Evosep). Connection to the Orbitrap Astral (Thermo) was established by an EASY‐Spray source (Thermo) at a spray voltage of 2000 V.

### 2.14 Mass spectrometry

We acquired our samples on the Orbitrap Astral in DIA mode. MS resolution was 240 000 with a scan range of 380−980 m/z and 500% normalised AGC target. Scanning was performed with 4‐Th isolation windows and a maximum IT of 5 ms. The isolated ions were further fragmented at 25% HCD collision energy. Field asymmetric ion mobility spectrometry was enabled (−40 compensation voltage, 2.5 L/min gas flow).

### 2.15 Raw data processing

We converted the raw to .mzml files using msconvert and searched library free with a predicted spectral library in DIA‐NN (v1.8.1; RRID:SCR_022865).^42^ UP000005640_9606 and UP000005640_9606_additional were searched (uniprot human databases) and the library contained 100 880 proteins and 20 491 genes. Variable modification was limited to methionine oxidation and a maximum of one missed cleavage was permitted. The following settings were used within DiaNN: peptide length range 7‒50, precursor charge 1‒5, precursor mass range 300‒1800 and fragment ion m/z range 200‒1800. Based on a prior estimation with five separate files, mass accuracy was fixed at 6, MS1 accuracy at 4 and scan window radius at 7 ppm for all samples. All other settings were left at default mode and MBR was enabled. FDR remained as per default at .01 and the ‘pg.matrix.tsv’ output was used for further analyses.

### 2.16 Bioinformatics analysis of MS data

Differential protein expression was analysed within R using ‘Limma v3.58.1’ (RRID:SCR_010943). We corrected for multiple testing using Benjamini–Hochberg, with an FDR of 5%. Volcano plot depict proteins that are differentially expressed, with an adjusted *p*‐value below .05 and logFC ±.5.

### 2.17 Cytokine array

A375 transfected cells (8 × 10^5^) were seeded in T75 culture flasks with 15 mL RPMI complete media. After 48 h, the supernatant was collected and centrifuged at 1000 rpm for 5 min. A 500 µL supernatant from each transfectant was analysed using the Proteome Profiler Human XL Cytokine Array Kit (R&D Systems, Cat# ARY022B) according to the manufacturer's protocol. Chemiluminescence was detected with the Sapphire Biomolecular Imager (Azure Biosystems) and optical densities were quantified using Image Lab Software 6.1. The global background subtraction method was applied to all the dots in the arrays and the software calculates the ‘adjusted volume’ after background subtraction. These values were normalised to the mean adjusted volume from the ‘reference spots’ of each membrane and then to the values measured in the control condition (empty vector), resulting in a FC value. The log_2_FC was calculated to represent the data in a heatmap.

### 2.18 Flow cytometry

After 24 h stimulation with or without 50 ng/mL interferon‐gamma (IFN‐γ, Shenandoah Biotechnology Cat# 100‐77‐20) and at a confluence of 80%, A375 transfected cells were trypsinised, centrifuged at 1000 rpm for 5 min and washed twice with ice‐cold DPBS. 1 × 10^6^ cells were labelled with Zombie NIR Fixable Viability Kit (1:2000; BioLegend Cat# 423105) and then treated with Human TruStain FcX (1:1000; BioLegend Cat# 422302). Cells were then surface stained with the following fluorochrome‐conjugated antibodies (1:200) for 30 min at 4°C in the dark: anti‐HLA‐ABC (clone W6/32) APC (eBioscience, Thermo Fisher Scientific Cat# 17‐9983‐42, RRID:AB_10733389), anti‐CD274 (clone B7‐H1, PD‐L1), PE (BioLegend Cat# 329705 [also 329706], RRID:AB_940366), anti‐HLA‐DR (clone L243) AF488 (BioLegend Cat# 307656 [also 307619, 307620], RRID:AB_2564168) and anti‐HLA‐DQ (clone HLADQ1) FITC (BioLegend Cat# 318104, RRID:AB_604128). Data acquisition was performed on FACSCanto (BD Biosciences) and analysed with FlowJo software v10 (BD Biosciences, RRID:SCR_008520).

### 2.19 RNA isolation, cDNA synthesis and real‐time quantitative PCR amplification

To quantify *CIITA* mRNA expression in the A375 transfected cells, total RNA was extracted from cell pellets containing approximately 4 × 10^6^ cells by using the RNeasy Mini Kit (Qiagen Cat# 74106), following the instructions outlined by the manufacturer. Next, the Revert Aid H Minus First Strand cDNA Synthesis Kit (Thermo Fisher Scientific Cat# K1632) was employed for the reverse transcription of RNA to cDNA. Real‐time quantitative PCR (RT‐qPCR) reactions were conducted on the QuantStudio 1 Real‐Time PCR System (Applied Biosystems). The reaction mixture was prepared by mixing 2 µL of cDNA samples with 6 µL of PowerTrack SYBR Green PCR Master Mix (Applied Biosystems Cat# A46112), .5 µL of forward and .5 µL of reverse primers (.3 µM) diluted in nuclease‐free water up to a total volume of 20 µL. Primer sequences: *CIITA*—forward: 5′‐CAAGTCCCTGAAGGATGTGGA‐3′, reverse: 5′‐ACGTCCATCACCCGGAGGGAC‐3′; *GAPDH*—forward: 5′‐TCAAGGCTGAGAACGGGAAG‐3′, reverse: 5′‐TGGACTCCACGACGTACTCA‐3′. Technical triplicates were performed for each biological sample. The data obtained from RT‐qPCR were analysed using the 2^‒ΔΔCT^ method. *CIITA* expression was normalised to the expression of the housekeeping gene *GAPDH*, followed by the use of the empty vector control to calculate the FC in expression.

### 2.20 Gene correlation analysis

To explore the correlation between *RECQL4* and MHC‐II‐related genes expression, we employed ‘Gene_Corr’ module of TIMER2.0. Spearman's correlations were calculated using the RNA‐seq data from the SKCM TCGA cohort.

### 2.21 Statistical analysis

R programming language (version 4.0.5) and GraphPad Prism version 9 (GraphPad Software, RRID:SCR_002798) were used for statistical investigation. Continuous variables were presented as median ± interquartile range and compared using Wilcoxon rank sum test for two variables or Kruskal‒Wallis test for more than two variables, unless otherwise stated. Categorical variables were analysed using two‐sided Fisher's exact test and chi‐square test when comparing two or more than two variables, respectively.

## 3 RESULTS

### 3.1 Prevalence of *RECQL4* high amplification in metastatic cancer sites correlates with decreased OS

**FIGURE 1:**
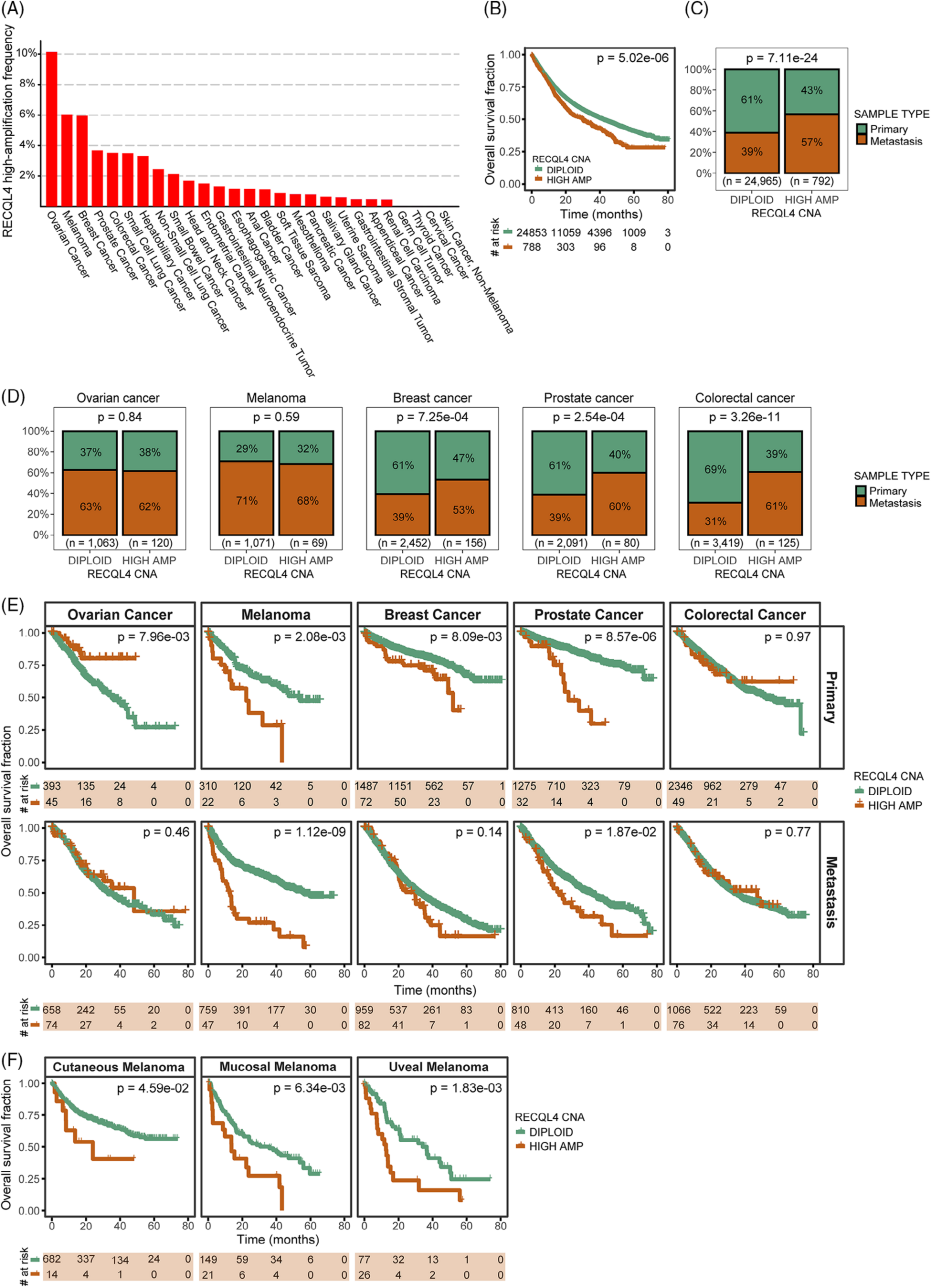
*R* *ECQL4* high amplification impacts disease progression and survival. (A) *RECQL4* high‐amplification frequency across different cancer entities in the Memorial Sloan Kettering—Metastatic Events and Tropisms (MSK‐MET) total cohort. (B) Kaplan‒Meier analysis for overall survival of cancer patients according to *RECQL4* copy number alteration (CNA). (C) Percentage of primary and metastatic tumour samples according to *RECQL4* CNA status in the total cancer cohort and (D) in the individual cancer cohorts. (E) Kaplan‒Meier overall survival analysis according to *RECQL4* CNA status in primary and metastatic tumour samples across cancer cohorts. (F) Kaplan‒Meier overall survival analysis according to *RECQL4* CNA status in the different melanoma subtypes. Cohort: MSK‐MET.^24^ Statistics: (B, E and F) log rank test and (C and D) two‐sided Fisher's exact test, *p* values as indicated; *p* < .05 was considered significant.

To investigate the impact of *RECQL4* gene amplification in different cancer entities, we analysed the MSK‐MET cohort^24^ where we detected a frequency of 3.07% (792 out of 25 775 cases) for *RECQL4* high amplifications. The highest frequency of *RECQL4* amplification was detected in ovarian cancer (10.14%; 120 of 1183 cases), melanoma (6.04%; 69 of 1142 cases), breast cancer (5.98%; 156 of 2609 cases), prostate cancer (3.68%; 80 of 2172 cases) and colorectal cancer (3.52%; 125 of 3548 cases) (Figure 1A). Moreover, patients presenting *RECQL4* high amplification showed reduced OS compared to patients with lack of *RECQL4* gene amplification (Figure 1B, *p* = 5.02e‐06). Furthermore, we detected a greater prevalence of *RECQL4* high amplification in metastatic lesions compared to primary tumours, but also a predisposition for *RECQL4* high amplification (57%) in metastases of multiple tumour entities compared to the diploid status (39%) (Figure 1C, *p* = 7.11e‐24). Next, we further explored the top five cancer cohorts with *RECQL4* high amplification (Figure 1A). Except for ovarian cancer and melanoma, analyses of the individual cancer cohorts revealed the dominance of *RECQL4* high amplification in metastatic samples over primary tumour tissues (Figure 1D, *p* < .05). In addition, we performed survival analyses in these cancer cohorts to investigate whether *RECQL4* high amplification correlated with patient's prognosis. Interestingly, we observed that the presence of *RECQL4* high amplification was associated with reduced OS in melanoma, breast cancer and prostate cancer (Figure 1E). Furthermore, high *RECQL4* amplification was consistently associated with shorter OS across different melanoma histological subtypes (cutaneous, mucosal and uveal melanoma, Figure 1F).

### 3.2 *RECQL4* high‐amplified samples present an increased FGA, but reduced microsatellite instability and TMB

**FIGURE 2:**
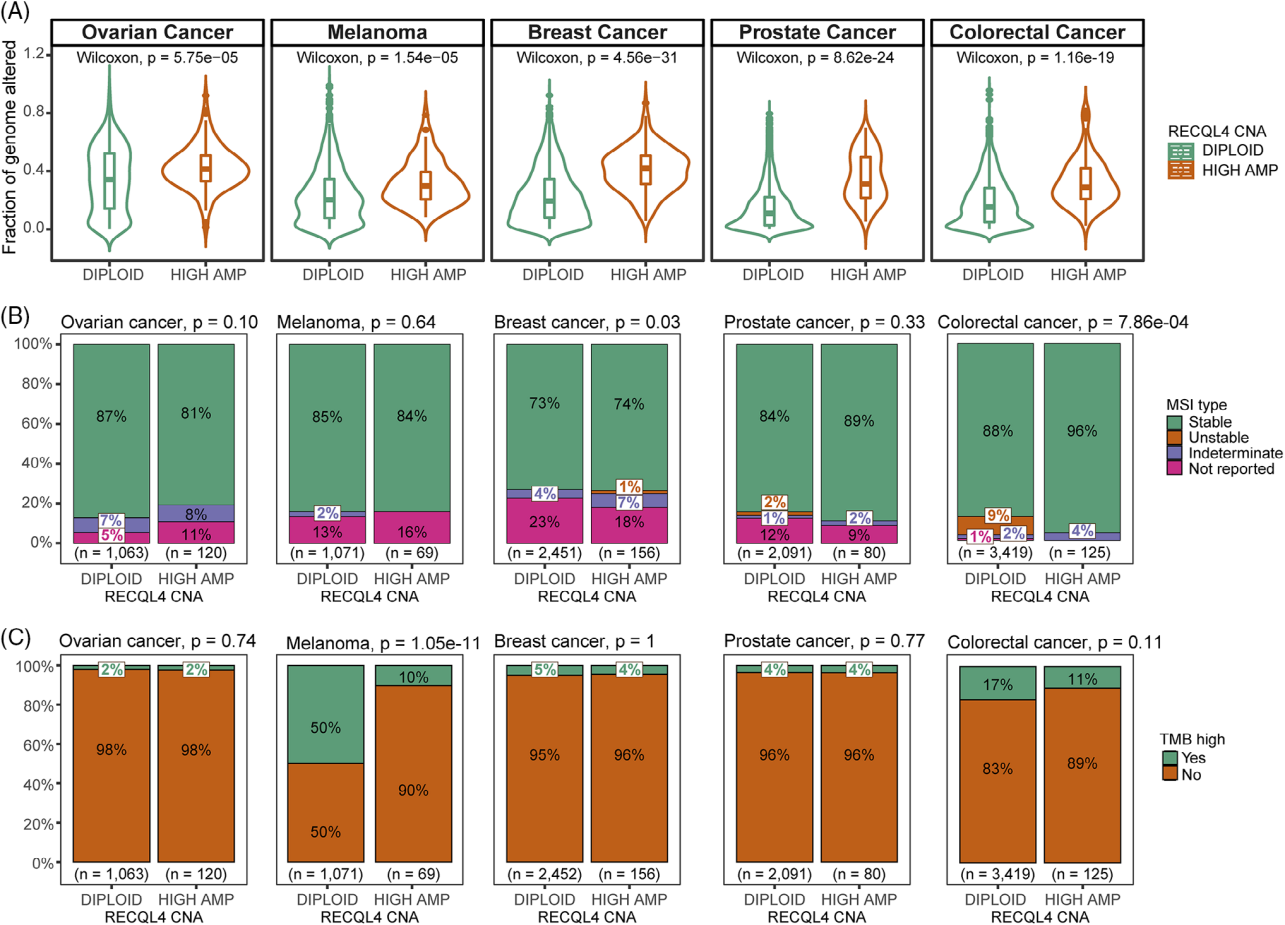
*RECQL4* high amplification is associated with higher fraction of genome altered (FGA), but reduced microsatellite instability (MSI) and tumour mutational burden (TMB). (A) FGA, (B) MSI type and (C) proportion of TMB high cases comparing diploid and high‐amplified samples for the *RECQL4* locus in ovarian cancer, melanoma, breast, prostate and colorectal cancers. Cohort: Memorial Sloan Kettering—Metastatic Events and Tropisms (MSK‐MET).^24^ Statistics: (A) Wilcoxon rank sum test, (B) chi‐squared test and (C) two‐sided Fisher's exact test were used to compare differences between the *RECQL4* diploid and high‐amplified group, *p*‐values as indicated; *p* < .05 was considered significant.

Interestingly, we noted an increased FGA in samples with elevated *RECQL4* amplification levels compared to those with a diploid status (Figure 2A, *p* < .05). FGA indicates the extent of genome disruption attributable to significant copy number variations, indicative of chromosomal instability (CIN). This CIN phenomenon has the potential to drive tumour growth, worsen patient prognosis, promote resistance to anticancer therapies,^43^, ^44^ and could be both, a causative factor or a consequence of the presence of *RECQL4* high amplification. Since *RECQL4* is implicated in DNA damage repair, we further analysed the relationship between *RECQL4* high amplifications and DNA damage repair alterations. We found that MSI was reduced in the colorectal cancer samples presenting *RECQL4* high amplifications (Figure 2B, *p* = 7.86e‐04). Furthermore, the proportion of melanoma samples with high TMB (≥10 mut/Mb) was diminished in the presence of *RECQL4* high amplification (Figure 2C, *p* = 1.05e‐11). High MSI and high TMB are both known as predictors for improved response to immune checkpoint therapy, especially in colorectal cancer and melanoma, presenting increased frequency of both events.^45^, ^46^ Thus, we propose that the increased FGA in these tumours induces *RECQL4* high amplification, leading to enhanced *RECQL4* expression for proficient DNA damage repair within the tumours. Consequently, this mechanism may reduce both MSI and TMB.

### 3.3 Suppression of immune‐related pathways contributes to the establishment of an immune‐evasive phenotype in the context of *RECQL4* high amplification

**FIGURE 3:**
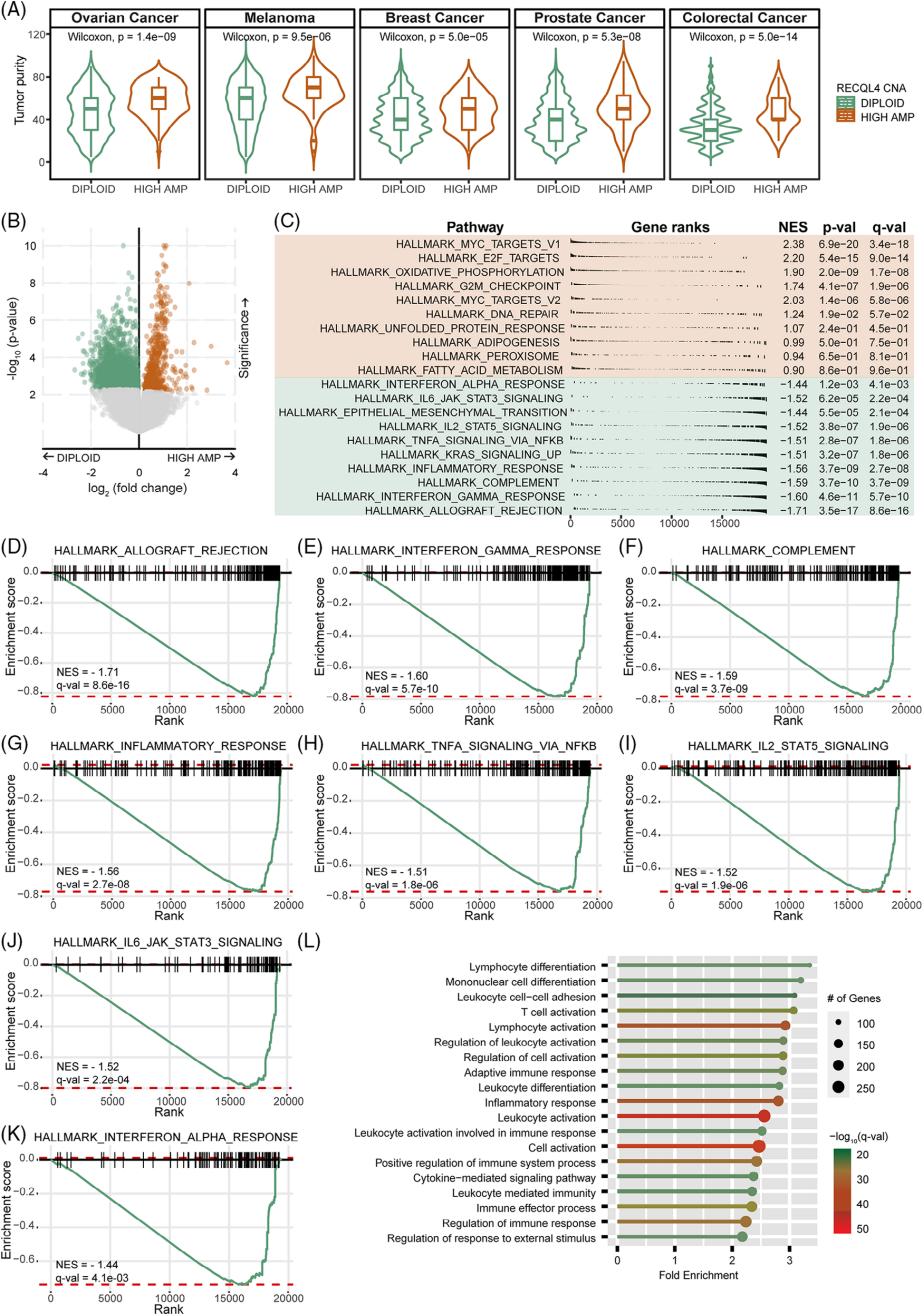
*RECQL4* high amplifications drive signatures of immune‐modulation. (A) Tumour purity compared between *RECQL4* diploid and high‐amplified samples in ovarian cancer, melanoma, breast, prostate and colorectal cancers. (B) Volcano plot showing the differentially expressed genes between *RECQL4* diploid versus high‐amplified melanoma samples. Brown and green dots represent significant genes (upregulated: brown, *n* = 490; downregulated: green, *n* = 1646) in *RECQL4* high‐amplified samples, compared to diploid samples. (C) Top 10 upregulated (brown) and downregulated (green) pathways of the Molecular Signatures Database (MSigDB) in the *RECQL4* high‐amplified samples compared to the diploid samples. Gene set enrichment analysis (GSEA) for individual immune‐related pathways involved in (D) allograft rejection, (E) interferon‐gamma (IFN‐γ) response, (F) the complement system, (G) inflammatory response, (H) nuclear factor kappa B (NF‐κB)‐driven tumour necrosis factor‐alpha (TNF‐α) signalling, (I) IL2‒STAT5 signalling, (J) IL6‒JAK‒STAT3 signalling and (K) IFN‐α response; presented in the order of significance. (L) Enriched gene ontology (GO) biological processes among the downregulated gene list from (B). The dot size denotes the number of genes and dot colour represents the –log10 (*q*‐value). Cohorts: (A) Memorial Sloan Kettering—Metastatic Events and Tropisms (MSK‐MET)^24^ and (B‒L) Skin Cutaneous Melanoma cohort from The Cancer Genome Atlas (SKCM TCGA). Statistics: (A) Wilcoxon rank sum test and (B) Student's *t*‐test were used to compare differences between the *RECQL4* diploid and high‐amplified groups. (C‒K) Pathway enrichment analyses results are presented by the normalised enrichment score (NES), *p*‐value (*p*‐val) and the false discovery rate (FDR) adjusted *p*‐value corrected by Benjamini–Hochberg multiple test (*q*‐value, *q*‐val); *q* < .05 was considered significant.

When analysing tissue characteristics of the MSK‐MET cohort,^24^ we identified a significantly increased level of tumour purity in tumours harbouring *RECQL4* high amplifications (Figure 3A, *p* < .05), implicating a larger fraction of cancer cells in the tumour mass. In light of these findings, we postulated that recruitment of cells from the tumour microenvironment, including cells of the immune compartment, might be constrained under conditions of elevated *RECQL4* copy number. To delineate the genes and pathways that are differentially regulated in tumours with *RECQL4* CNA, we grouped samples of the TCGA SKCM cohort according to the *RECQL4* CNA status (diploid and high‐amplified) and performed gene expression analyses. We detected a total of 2136 significantly differentially expressed genes, of which 490 were upregulated and 1646 were downregulated in the samples corresponding to *RECLQ4* high amplification compared to diploids (Figure 3B). According to the GSEA, strong activation of pathways in samples presenting *RECQL4* high amplification were related to cell proliferation, cell cycle and DNA damage repair (MYC targets variant 1 and variant 2, E2F targets, G2M checkpoint and DNA repair) (Figure 3C). These pathways are closely linked to *RECQL4* intrinsic function. Besides, *RECQL4* high‐amplified samples also had increased metabolic activity (oxidative phosphorylation). This may be due to the expression of *RECQL4* in mitochondria, which is responsible for maintaining mitochondrial DNA integrity and coping with oxidative stress.^47^, ^48^ Of particular note, we observed a pronounced downregulation of key signalling pathways associated with the immune response in samples exhibiting high *RECQL4* amplification. These included pathways involved in allograft rejection, IFN‐γ and ‐α responses, the complement cascade, global inflammatory response, tumour necrosis factor‐alpha signalling via nuclear factor kappa B, IL2‒STAT5 signalling and IL6‒JAK‒STAT3 signalling (Figure 3D‒K). Moreover, employing GO Biological Process annotation, we uncovered a significant downregulation in pathways associated with lymphocyte differentiation or activation within samples exhibiting *RECQL4* high amplification (Figure 3L). Intriguingly, extending our analysis to a cohort of patients undergoing ICI treatment, consistent findings emerged.^27^ A total of 2180 genes exhibited significant differential expression, with 132 upregulated and 2048 downregulated in *RECQL4* high‐amplification samples compared to their diploid counterparts (Figure S1A). Further exploration through GSEA unveiled a confluence of downregulated pathways predominantly linked to immune responses (Figure S1B‒I), with notable attenuation observed in allograft rejection and the complement cascade, mirroring findings from the SKCM TCGA cohort. Additionally, the impairment in lymphocyte differentiation and activation was evident in the presence of *RECQL4* amplifications (Figure S1J). These findings suggest that there is a global decline in immune response in the presence of *RECQL4* high amplification that persists even under ICI treatment regimens.

### 3.4 *RECQL4* high amplification limits intra‐tumoural immune cell recruitment

**FIGURE 4:**
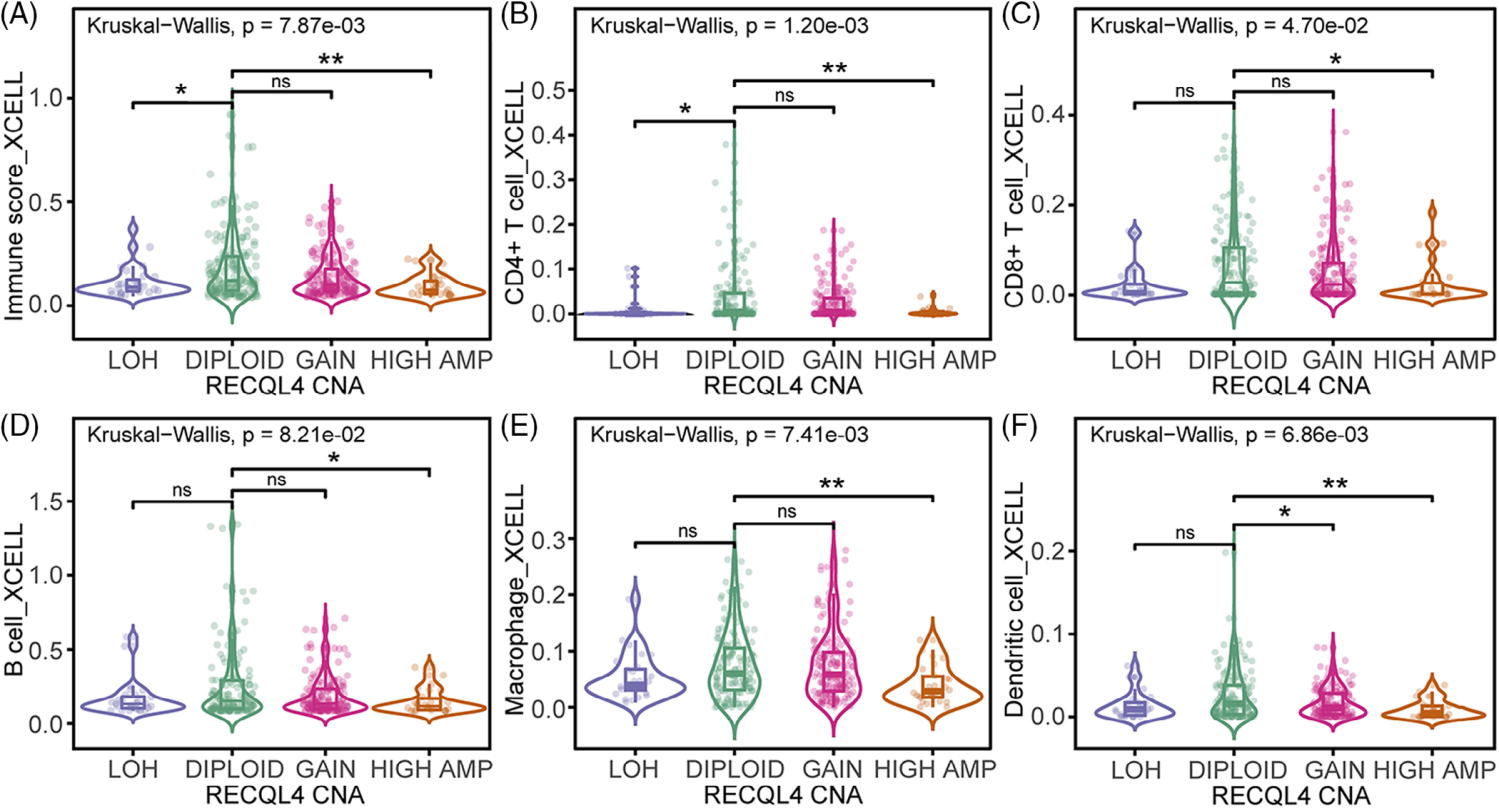
High *RECQL4* amplification is associated with reduced intra‐tumoural immune cell infiltration. (A) Immune score and intra‐tumoural infiltration by (B) CD4^+^ T cells, (C) CD8^+^ T cells, (D) B cells, (E) macrophages and (F) dendritic cells, comparing the different *RECQL4* copy number alterations (CNA): loss of heterozygosity (LOH), diploid, gain and high amplification (HIGH AMP). Cohort: Skin Cutaneous Melanoma cohort from The Cancer Genome Atlas (SKCM TCGA). Statistics: the xCell algorithm^39^ was used to calculate intra‐tumoural infiltration based on RNA‐seq data. Kruskal‒Wallis test was used to compare the different *RECQL4* copy number levels, *p*‐values as indicated (^*^ *p* < .05; ^**^ *p* < .01; ns, *p* > .05), *p* < .05 was considered significant.

The potential association between *RECQL4* high amplification and the host immune response in cancer patients captured our interest, prompting us to compares intra‐tumoural immune cell infiltration across various *RECQL4* CNA within the TCGA SKCM cohort. This analysis encompassed distinct categories including LOH, diploid status, copy number gain and high amplification. By quantifying the immune score, our results showed impaired intra‐tumoural infiltration of immunoreactive cells in samples with *RECQL4* high amplification compared to diploid samples (Figure 4A, *p* = 5e‐03). Next, we asked which immune cells are targeted by *RECQL4* CNA for its presentation in tumour tissue. We focused our analyses on T‐cell subtypes (Figure 4B,C), B cells (Figure 4D), macrophages (Figure 4E) and dendritic cells (Figure 4F), all known for their key role in shaping the response to cancer immunotherapy. Strikingly, we observed reduced numbers of all immune cell types in the samples with *RECQL4* high amplifications (Figure 4B–F). Thus, *RECQL4* high amplification may affect patient prognosis due to the limited number of immune cells and modulation of immune‐related pathways in these tumours.

### 3.5 *RECQL4* dampens clinical response to ICI and acts as a reliable independent prognostic factor for survival in melanoma patients

**FIGURE 5:**
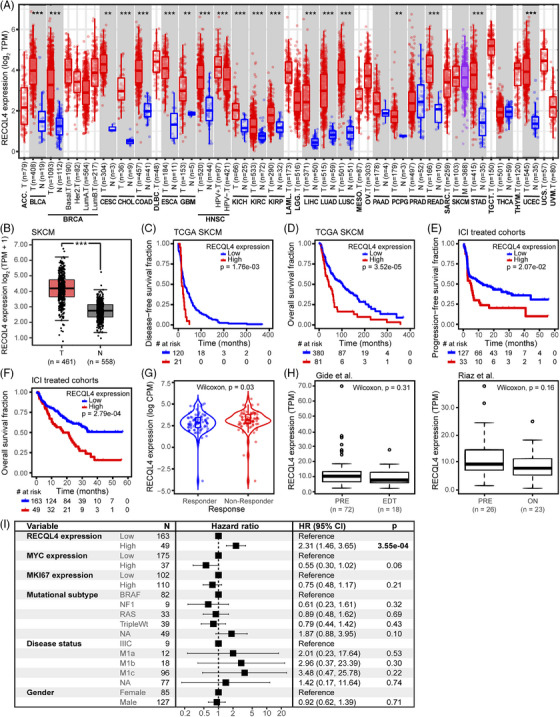
*RECQL4* impacts survival and immune checkpoint inhibitor (ICI) response and is an independent prognostic factor in melanoma patients. (A) *RECQL4* expression levels (log_2_ transcript per millions [TPM]) across cancer entities grouped according to sample type: tumour (red), healthy (blue) and metastatic (purple) tissues. (B) *RECQL4* expression levels (log_2_ TPM+1) comparing melanoma tumour (Skin Cutaneous Melanoma cohort from The Cancer Genome Atlas [TCGA SKCM]) and normal skin tissues (Genotype‐Tissue Expression [GTEx]). (C) Disease‐free, (E) progression‐free and (D and F) overall survival fraction comparing *RECQL4* low‐ and high‐expression groups. (G) *RECQL4* expression levels (log counts per millions [CMP]) comparing responders and non‐responders. (H) *RECQL4* expression levels (TPM) in samples obtained prior‐therapy (PRE) and early during treatment (EDT, 7−14 days following the initiation of immunotherapy) or on‐treatment (ON, 23−29 days following the initiation of immunotherapy). (I) Forest plot for multivariate Cox proportional hazards (CoxPH) model. Each variable is displayed in a row and the corresponding number (*N*) of samples, hazard ratio (HR) with 95% confidence intervals (CI, in brackets), and *p*‐values are indicated. *p* < .05 from a log rank test was considered significant and marked in bold. Cohorts: (A) pan‐cancer cohort from TCGA including 33 cancer types; (B) SKCM cohort from TCGA and healthy skin data from GTEx; (C and D) SKCM TCGA; (E‒G, I) ICI‐treated melanoma cohort; (H, left) Gide et al.^29^ and (H, right) Riaz et al.^30^ Statistics: (A and B) Wilcoxon rank sum test, *p*‐values as indicated (^*^ *p* < .05; ^**^ *p* < .01; ^***^ *p* < .001; ns, *p* > .05). (C‒F) Log rank test, *p* < .05 was considered significant. (G and H) Wilcoxon rank sum test, *p*‐values as indicated. Abbreviations of the cancer types presented in (A) can be found at https://gdc.cancer.gov/resources‐tcga‐users/tcga‐code‐tables/tcga‐study‐abbreviations.

So far, we have demonstrated the pan‐cancer role of *RECQL4* CNA. The subsequent objective was to ascertain whether *RECQL4* transcriptional alterations also play a role in melanoma. As expected, *RECQL4* gene expression was significantly increased with *RECQL4* copy number gain or high amplification compared to diploid genotypes (Figure S2A, *p* < .0001) in the TCGA SKCM cohort. Interestingly, similar trends were observed in an independent cohort of ICI‐treated melanoma patients (Figure S2B, *p* = .02). To test our hypothesis that *RECQL4* upregulation may be associated with cancer progression per se, we analysed *RECQL4* expression comparing tumour samples to adjacent normal tissue in several tumour entities from the TCGA database. Apart from pancreatic adenocarcinoma, prostate adenocarcinoma and thyroid carcinoma, we found a significant upregulation of *RECQL4* expression in tumours from 18 out of 21 cancer entities (Figure 5A, *p* < .01). Since for SKCM only expression data from tumour and metastatic tissue are available in the TCGA database, we also used GTEx data for normal tissue from the GEPIA2 web tool to compare *RECQL4* expression in tumour versus healthy skin and in this case observed a significant upregulation of *RECQL4* driven by tumour development (Figure 5B, *p* < .001).

Next, we investigated the potential correlation between *RECQL4* expression and patient survival. High *RECQL4* expression was associated with significantly shorter DFS (*p* = 1.76e‐03) and OS (*p* = 3.52e‐05) in the TCGA SKCM cohort (Figure 5C,D). Interestingly, high baseline *RECQL4* expression was also predictive of shorter PFS (*p* = 2.07e‐02) and OS (*p* = 2.79e‐04) in melanoma patients receiving immune checkpoint‐targeted therapy (Figure 5E,F). We also clustered patients according to the Response Evaluation Criteria In Solid Tumours (RECIST) for their response to ICI therapy. Of note, biopsies from patients who did not respond to ICI treatment (non‐responder, RECISTv1.1 progressive disease) presented elevated *RECQL4* expression level when compared to responders (RECISTv1.1 complete response or partial response) (Figure 5G, *p* = .03). Finally, we asked if the immune checkpoint blockade treatment per se is able to induce a shift in *RECQL4* expression in melanoma patients. For that, we chose the Gide et al.^29^ and Riaz et al.^30^ cohorts, as they include RNA‐seq data from melanoma patient biopsies prior and on‐treatment/after ICI‐directed therapy. Both cohorts confirmed that there was no shift following therapy onset, underscoring the importance of baseline *RECQL4* helicase levels (Figure 5H).

Our data have clearly identified an association of *RECQL4* with disease progression and shorter survival in melanoma patients. Nevertheless, we sought to test the power of *RECQL4* as an independent prognostic factor. Employing CoxPH model analysis, we concurrently evaluated the impact of multiple clinical and genomic variables on survival duration within an ICI‐treated melanoma cohort. In addition to *RECQL4* expression, our variables of interest included *MKI67* (marker of proliferation Ki‐67) and *MYC* (MYC Proto‐Oncogene, BHLH transcription factor) expression, recognised for their roles as proliferation markers and oncogenic drivers, respectively. We also incorporated various clinical variables deemed relevant to survival based on available clinical data. *MKI67* expression, being a well‐established proliferation marker,^49^ was specifically chosen due to its association with highly proliferative tumours typically associated with worse prognosis. Confirming *RECQL4*’s independence from *MKI67* as a prognostic factor would imply that *RECQL4*’s influence on patient prognosis extends beyond tumour proliferation rate. Furthermore, considering the physical proximity of the *MYC* oncogene to the *RECQL4* gene locus, coupled with *MYC*’s established impact on tumour progression and immune evasion,^50^ we included *MYC* expression as a variable in our multivariate analyses. Remarkably, our analyses revealed *RECQL4* expression as an independent prognostic factor associated with poor prognosis (HR > 1) (Figure 5I, *p* = 3.55e‐04). Intriguingly, neither the expression level of *MKI67* and *MYC* nor the clinical variables or mutational subtypes per se demonstrated independent prognostic significance.

### 3.6 RECQL4 favours an immunosuppressive tumour microenvironment by downregulating MHC‐II molecules and key immune‐regulatory factors

**FIGURE 6:**
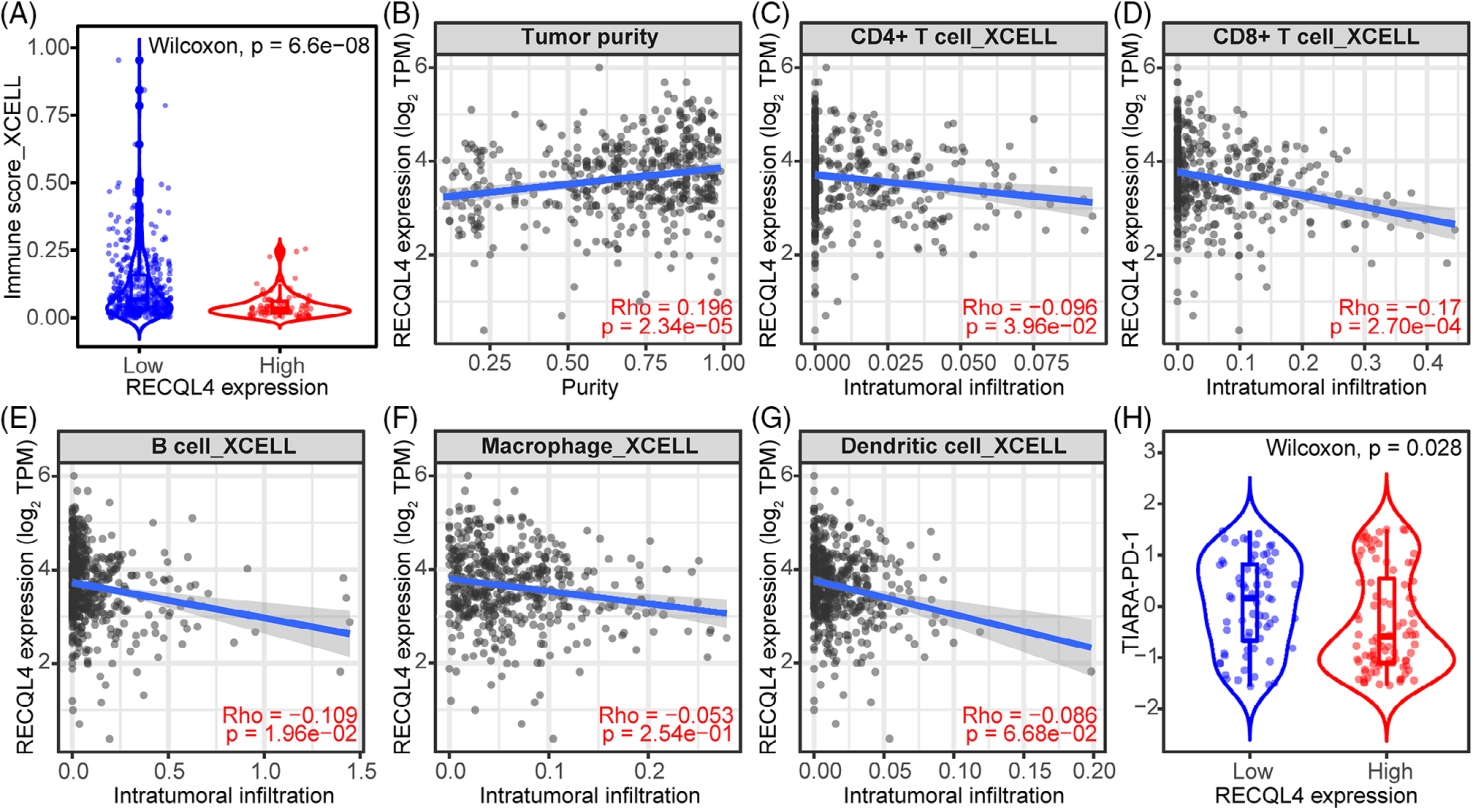
*RECQL4* expression levels direct immune cell recruitment and tumour immunogenicity associated with response to anti‐PD‐1 (TIARA‐PD‐1) signature. (A) Immune score comparing low and high *RECQL4* expression. (B) Correlation of *RECQL4* expression and tumour purity. Correlation of *RECQL4* expression and infiltration level of (C) CD4^+^ T cells, (D) CD8^+^ T cells, (E) B cells, (F) macrophages and (G) dendritic cells. (H) TIARA‐PD‐1 comparing low and high *RECQL4* expression levels. Cohorts: (A–G) Skin Cutaneous Melanoma cohort from The Cancer Genome Atlas (SKCM TCGA) and (H) ICI‐treated melanoma cohort. Statistics: (A–G) the xCell algorithm^39^ was used to calculate intra‐tumoural infiltration based on RNA‐seq data. (H) TIARA‐PD‐1 values were obtained from Campbell et al.^40^ (A and H) Wilcoxon rank sum test. (B) Spearman's rank correlation (rho). (C–G) Spearman's rank correlation (rho) adjusted for tumour purity.

Having identified the robust association of RECQL4 to ICI resistance, we next explored the immunogenic landscape in this context. Samples exhibiting high *RECQL4* expression displayed significantly lower immune scores (Figure 6A, *p* = 6.6e‐08) and higher tumour purity (Figure 6B, *p* = 2.34e‐05). These findings were further substantiated by the negative correlation observed between *RECQL4* expression and the intra‐tumoural abundance of CD4^+^ and CD8^+^ T cells (Figure 6C,D, *p* = 3.69e‐02 and 2.70e‐04), B cells (Figure 6E, *p* = 1.96e‐02), macrophages (Figure 6F, *p* = 2.54e‐01) and dendritic cells (Figure 6G, *p* = 6.68e‐02). In an effort to validate our hypothesis regarding the potential impact of RECQL4‐mediated limitation of intra‐tumoural targets on ICI therapy response, we sought to replicate these findings in patients undergoing ICI treatment. Consequently, we observed a reduction in the TIARA‐PD‐1 score in samples exhibiting high *RECQL4* expression (Figure 6H, *p* = .028), providing further evidence of the potential influence of *RECQL4* expression on the efficacy of ICI therapy.

To elucidate the mechanistic principle of RECQL4's involvement in our observed phenomena and its potential interference with ICI resistance in melanoma, we performed gain and loss of function analyses by generating genetic variants. We verified an up to threefold increased protein expression in the A375 transfectants expressing RECQL4 or the mutant RECQL4^K508A^ (Figure S3A, *p* = .0115 and .0022) when compared to the empty vector control. RECQL4^K508A^ exhibits a loss of helicase and ATPase activity,^51^ while still retaining DNA binding and strand annealing activity.^52^ We confirmed the decreased activity of RECQL4^K508A^ by measuring HRR levels in the cells, which were strongly increased upon RECQL4 overexpression, but only slightly altered when RECQL4^K508A^ was expressed (Figure S3B).

**FIGURE 7:**
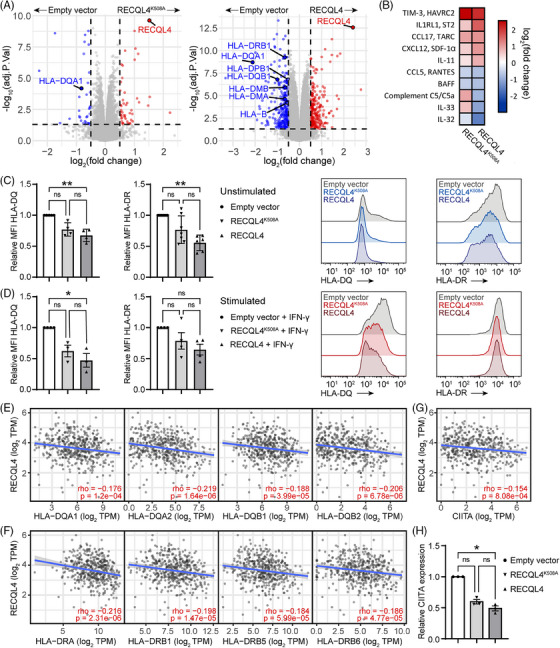
RECQL4‐driven downregulation of major histocompatibility complex class II (MHC‐II) molecules and cytokines involved in immune cell recruitment and activity. (A) Volcano plots showing the differentially expressed proteins between RECQL4^K508A^ (left) and RECQL4 (right) transfected A375 melanoma cells and the empty vector control. Red and blue dots indicate proteins significantly upregulated and downregulated, respectively. (B) Heatmap showing the fold change in protein expression of soluble factors secreted by the individual transfectants compared to empty vector control. (C and D) Relative HLA‐DQ and HLA‐DR median fluorescence intensity (MFI) (left) and representative flow cytometry histogram showing the fluorescence intensity (log_10_ scale) of HLA‐DQ and HLA‐DR (right) in the RECQL4‐overexpressing cells compared to the empty vector control (C) without and (D) with interferon‐gamma (IFN‐γ) stimulation during 24 h. (E) Correlation of *RECQL4* expression and *HLA‐DQA1*, *HLA‐DQA2*, *HLA‐DQB1* and *HLA‐DQB2* mRNA expression. (F) Correlation of *RECQL4* expression and *HLA‐DRA*, *HLA‐DRB1*, *HLA‐DRB5* and *HLA‐DRB6* mRNA expression. (G) Correlation of *RECQL4* expression and *CIITA* mRNA expression. (H) Relative *CIITA* expression measured by real‐time quantitative PCR (RT‐qPCR) in the A375 transfected cell lines. Statistics: (C, D and H) Bar plots represent the mean ± SEM (*n* = 4 in C left, *n* = 7 in C right, *n* = 3 in D left, *n* = 4 in D right, *n* = 3 in H). The results were analysed using Kruskal‒Wallis and Dunn's post hoc multiple comparison test. *p*‐Values as indicated (^*^ *p* < .05; ^**^ *p* < .01; ns, *p* > .05), *p* < .05 was considered significant. (E–G) Spearman's rank correlation (rho), *p*‐values as indicated. Cohort: Skin Cutaneous Melanoma cohort from The Cancer Genome Atlas (SKCM TCGA).

To obtain a broader view of the alterations driven by RECQL4 overexpression, we performed MS‐based proteomics and were able to quantify 10 262 proteins with excellent data completeness (98.2% > 75% valid values) followed by calculation of the differential protein expression between RECQL4^K508A^ or RECQL4 transfected cells and the empty vector control. At an adjusted *p*‐value below .05 and a log_2_FC threshold of ±.5, we identified 48 upregulated and 33 downregulated proteins in the RECQL4^K508A^ transfectant, as well as 216 upregulated and 250 downregulated proteins in the RECQL4 transfectant, relative to the empty vector control. Notably, the expression levels of several MHC‐II molecules, including HLA‐DMA, HLA‐DMB, HLA‐DPB1, HLA‐DQA1, HLA‐DQB1 and HLA‐DRB1, were decreased upon RECQL4 overexpression compared to the empty vector control. In addition, HLA‐DQA1 was downregulated in the RECQL4^K508A^ transfectant (Figure 7A). Given the established association between MHC‐II expression and enhanced T‐cell tumour infiltration, as well as its prognostic value for response to ICIs in melanoma^53^ our findings suggest a potential linkage between RECQL4 and its immunomodulatory effects.

Additionally, our investigation encompassed an examination of the secretome derived from transfected cells in relation to the empty vector control, with the aim of elucidating the influence of secreted factors on the tumour microenvironment of RECQL4‐overexpressing melanomas. While many chemokines and cytokines possess dual roles necessitating further exploration of their role in anti‐tumour immunity, we observed an augmentation of soluble factors known for their tumourigenic and immune‐suppressive roles, such as TIM‐3, IL1RL1, CCL17, CXCL12 and IL‐11, in the supernatants of the transfected cells compared to the control. These factors predominantly favour regulatory T‐cell infiltration or macrophage M2 polarisation, thereby promoting tumour immune escape.^54^, ^55^, ^56^, ^57^, ^58^ Furthermore, we noted a decrease in the secretion of cytokines promoting immune cell activation or recruitment, including IL‐32, IL‐33, BAFF and CCL5,^59^, ^60^, ^61^, ^62^ as well as the complement C5a, by RECQL4‐overexpressing cells (Figure 7B).

In light of recent research emphasising the critical role of MHC II‐dependent CD4^+^ T‐cell immunity in combating cutaneous melanoma,^63^, ^64^ we deeper analysed the interplay between RECQL4 and MHC‐II‐associated molecules. Employing flow cytometry analysis, we discovered that surface expression of HLA‐DQ and HLA‐DR is diminished upon RECQL4 overexpression, irrespective of IFN‐γ stimulation (Figure 7C,D). Notably, this downregulation is specific to MHC‐II‐related molecules, as evidenced by the unaltered expression of MHC‐I‐related molecules (HLA‐ABC) and PD‐L1 surface markers in RECQL4‐overexpressing cells (Figure S4). In line with these findings, we observed a concomitant reduction in the transcription of genes encoding MHC‐II‐related molecules (*HLA‐DQA1*, *HLA‐DQA2*, *HLA‐DQB1*, *HLA‐DQB2*, *HLA‐DRA*, *HLA‐DRB1*, *HLA‐DRB5* and *HLA‐DRB6*) upon *RECQL4* overexpression in human tissues (Figure 7E,F). Finally, we have conclusively identified the class II major histocompatibility complex transactivator (CIITA) as the central mechanistic mediator linking RECQL4 to MHC‐II expression. Through comprehensive analysis, we unveiled a significant inverse relationship between *RECQL4* and *CIITA* mRNA expression levels (Figure 7G, *p* = 8.08e‐04), corroborating this finding through the utilisation of genetically modified RECQL4 variants (Figure 7H).

**FIGURE 8:**
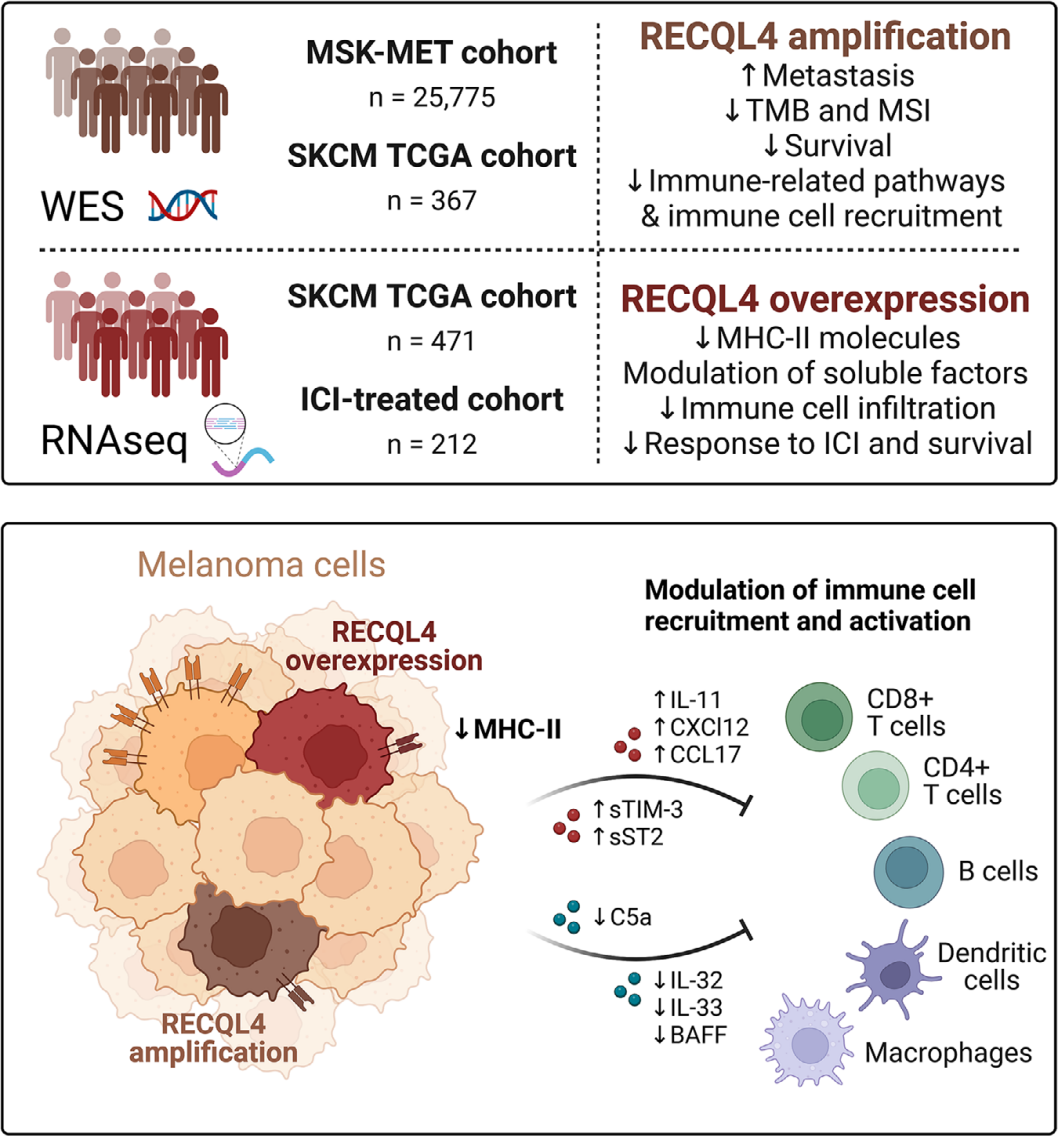
Schematic representation of the impact of *RECQL4* amplification and overexpression in melanoma. In the analysed cohorts, *RECQL4* amplification is associated with increased metastasis and poor survival rates in addition to decreased tumour mutational burden (TMB) and microsatellite instability (MSI). In addition, high RECQL4 levels were linked to the suppression of immune‐related pathways and immune cell recruitment, probably due to the reduced expression of major histocompatibility complex class II (MHC‐II) molecules and the modulation of immune‐related soluble factors. All these observations lead to poor response to immune checkpoint inhibitors (ICI) and worse overall survival in patients with high *RECQL4* expression.

In conclusion, the collective downregulation of MHC‐II expression and the alteration of immune‐related soluble factors driven by RECQL4 may promote tumour evasion, potentially impairing the efficacy of ICI therapy in melanoma patients (Figure 8).

## 4 DISCUSSION

The DDR is a fundamental process for maintaining genome integrity, and its dysfunction is a defining characteristic of cancer DNA damage often occurs as a result of exogenous and endogenous factors such as ultraviolet irradiation, errors in cell replication or oxidative stress. Defects in the DDR have been exploited therapeutically in cancer patients treated with ionising radiation or genotoxic chemotherapies. Although molecular targeting of the DDR and/or immune checkpoints offer new hope for a cure in a wide range of cancers, clinical studies have shown that only a minority of patients benefit from this kind of therapeutic strategy. Moreover, a majority of ICI‐treated cancer patients still either develop adaptive resistance and relapse, or the therapy must be discontinued due to the rate of immune‐related adverse events. Besides the expression of immune checkpoint molecules, recent data indicate the impact of specific DDR gene alterations such as HRR deficiency, TMB‐high, dMMR or MSI for its significance towards ICI therapy response.^65^ Still, there is a lack of mechanistic understanding for targeting genome stability in the context of ICI therapy.

RecQ helicases, such as *RECQL4*, are part of the DDR system. Although recent work by others has correlated loss or gain of *RECQL4* expression with cancer susceptibility, the reciprocal interplay of *RECQL4*‐driven signalling pathways with signatures driving immunotherapy efficacy is yet to be elucidated. Now, using RNA‐seq, whole exome sequencing and clinical data from cancer patients, including several already published cohorts of melanoma patients under immunotherapy, our data indicate *RECQL4* as a potentially ‘druggable’ factor associated with an unfavourable anti‐tumour immune response. Recent work by Grasso et al. emphasised the relationship between elevated IFN‐γ signalling and improved response to ICI therapy through transcriptome analyses of biopsies from melanoma patients prior to‐ and on‐treatment.^66^ In light of these clinical data, *RECQL4* amplification/expression is of interest given the observed inverse relationship between IFN‐γ pathway activity and *RECQL4* expression levels. Interestingly, our data clearly show that *RECQL4* high amplification and expression correlated with downregulation of immune‐related pathways and reduced intra‐tumoural recruitment of immune cells, including T cells. In melanoma cells, IFN‐γ signalling has been demonstrated to drive antigen presentation and the release of chemokines that recruit T cells (such as CXCL9 and CXCL10). The preceding observations offer an explanation as to why the disruption of the IFN‐γ pathway may result in the development of resistance to immunotherapy.^67^ In line with this, poor prognosis and limited response to ICI are the suggested consequences of high *RECQL4* activity in our study.

Mechanistically, our gain and loss of function analyses have unveiled a notable downregulation of major MHC‐II molecules following RECQL4 overexpression, concomitant with alterations in the secretion of immune‐regulatory factors that foster an immunosuppressive tumour microenvironment. Moreover, our observations suggest the implication of RECQL4 in signalling pathways (e.g., IFN‐γ, JAK/STAT) modulating the induction of CIITA, the master regulator of MHC‐II expression, thereby contributing to the diminished expression of MHC‐II‐associated molecules. The combined effect of RECQL4‐induced changes in soluble factor secretion and MHC‐II downregulation likely facilitates tumour immune evasion, potentially elucidating the attenuated response to ICI therapy in patients exhibiting elevated RECQL4 expression. Nevertheless, further investigation into the reciprocal interplay between RECQL4 and MHC‐II‐mediated immune tolerance is imperative, as this domain remains largely unexplored in the current literature. Leveraging preclinical modelling approaches holds promise for providing invaluable insights into the direct regulatory role of RECQL4 on MHC‐II and its implications for mechanisms governing immune tolerance.

Given the demonstrated roles of RECQL4 in cancer, it is logical to assume that therapeutic targeting may be an effective strategy for cancer therapy. Moreover, our findings support the concept that the downregulation of RECQL4 would enhance the recruitment and infiltration of immune cells, thereby presenting a potential therapeutic target for immune checkpoint‐mediated therapy. In addition, RECQL4 plays a dual role in DNA replication and repair mechanisms in normal and tumour cells. Since tumour cells display enhanced proliferation relative to most normal cells, with the exception of progenitor and stem cells, targeting the replication machinery through RECQL4 seem to be a promising strategy with limited consequences for non‐cancerous cells. Of note, most recent data show a positive correlation between *RECQL4* expression and stem cell markers like CD133, Nestin and Musashi, indicating the critical impact of *RECQL4* on stemness.^22^ In addition, mice show enhanced apoptotic cell death in multipotent progenitor cells lacking *RECQL4*‐expression relative to wild‐type.^68^ Hence, targeting of RECQL4 may constitute an effective strategy to prevent cancer recurrence through depletion of cancer stem cells. Regarding the therapeutic targeting of RECQL4, there are currently two drugs under investigation. The antibiotic Heliquinomycin, was shown to inhibit some of the DNA replicative helicases, including RECQL4.^69^ Additionally, the histone deacetylase inhibitor trichostatin A led to suppression of RECQL4 in prostate cancer cells.^14^

Therefore, there are ongoing efforts to identify different histone deacetylase inhibitors for therapeutic RECQL4 targeting. We believe indirect targeting of RECQL4 to be far less specific than direct inhibition with RECQL4 inhibitors, quite certainly leading to more side effects. Thus, screening, identification and optimisation of selective and specific RECQL4 inhibitors is warranted to avoid unclear pharmacology and side effects. In addition, more insights in RECQL4‐driven signalling pathways in large patient cohorts under ICI therapy are needed to better understand the impact of REQCL4 on cancer‐related modulators that influence disease progression and ICI response. It is therefore imperative to conduct thorough and meticulous research into these issues prior to the introduction of RECQL4 inhibition in melanoma patients undergoing treatment with ICIs.

## 5 CONCLUSION

In conclusion, our comprehensive analysis sheds light on the complex interplay between RECQL4 amplification, genomic instability, immune modulation and clinical outcomes in cancer. The prognostic significance of RECQL4 alterations, coupled with its potential role as a predictive biomarker for immunotherapy response, underscores the clinical relevance of targeting RECQL4 in cancer therapy. A deeper understanding of the molecular mechanisms by which RECQL4 regulates the immune system may facilitate the development of innovative therapeutic strategies to enhance the clinical efficacy of immunotherapy in cancer patients.

## AUTHOR CONTRIBUTIONS

Iris Helfrich conceptualised, coordinated and directed the project. Sara Egea‐Rodriguez, Thierry M. Nordmann and Restuan Lubis performed experimental work. Sara Egea‐Rodriguez, Renáta Váraljai, Manuel Philip and Susanne Horn performed statistical analysis. Raphael Stoll performed binding analyses. Alexander Roesch, Florian Rambow, Fang Zhao, Annette Paschen, Susanne Horn, Ian D. Hickson and Dirk Schadendorf provided expertise and intellectual support for the project. Sara Egea‐Rodriguez, Renáta Váraljai and Iris Helfrich wrote the manuscript, and all authors edited and approved it.

## CONFLICT OF INTEREST STATEMENT

The authors declare they have no conflicts of interest.

## ETHICS STATEMENT

Informed patient consent was obtained from all patients. The study was performed with approval by the ethics committee of the Medical Faculty, University Duisburg‐Essen (ethics approval nos. 11‐4715 and 17‐7708‐BO).

## Supporting information



Supporting Information

Supporting Information

Supporting Information

Supporting Information

## Data Availability

The datasets analysed during the current study are available in the cBioPortal for Cancer Genomics (https://www.cbioportal.org/) and PhenoTImE (https://doc.hornlab.org/shiny/cru337phenotime/) online repositories.
