# Supplementary figures and images for: RECQL4 affects MHC class II‐mediated signalling and favours an immune‐evasive signature that limits response to immune checkpoint inhibitor therapy in patients with malignant melanoma
Source: Clin Transl Med. 2025 Jan 15;15(1):e70094. doi: 10.1002/ctm2.70094 (PMC11734436; doi:10.1002/ctm2.70094)

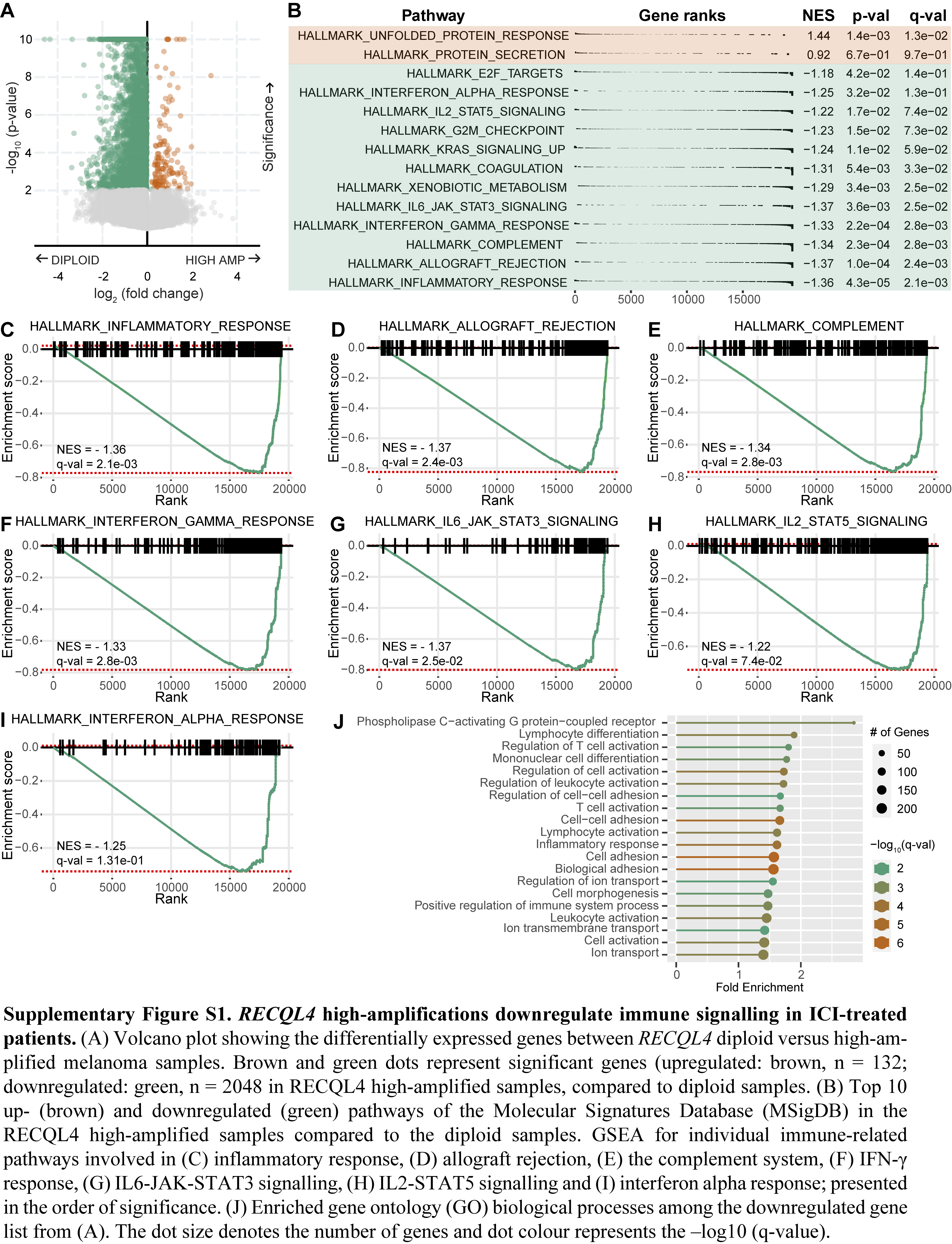

Supplement: Supplementary file 1 — Supporting Information [file CTM2-15-e70094-s002.tif]

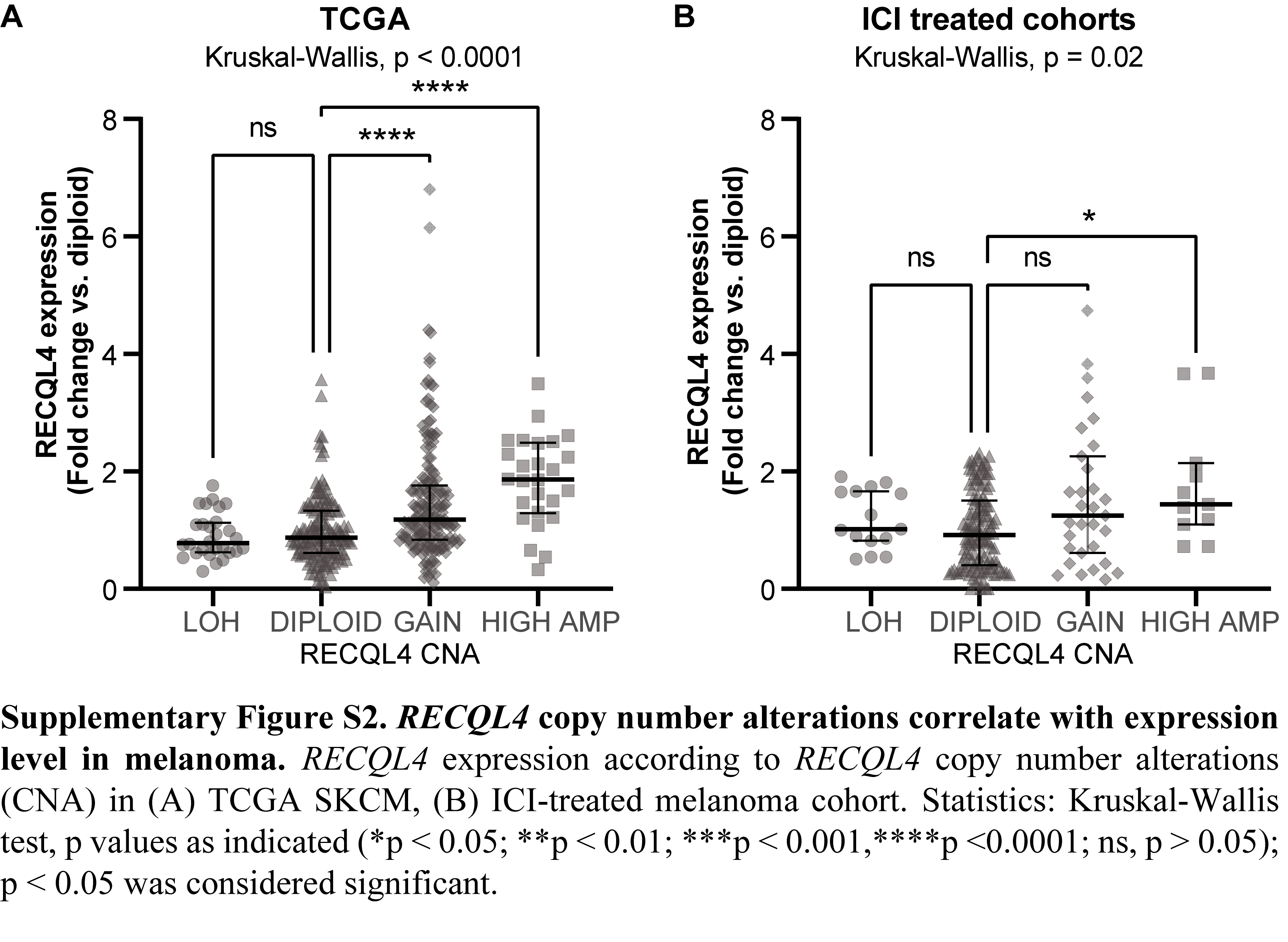

Supplement: Supplementary file 2 — Supporting Information [file CTM2-15-e70094-s003.tif]

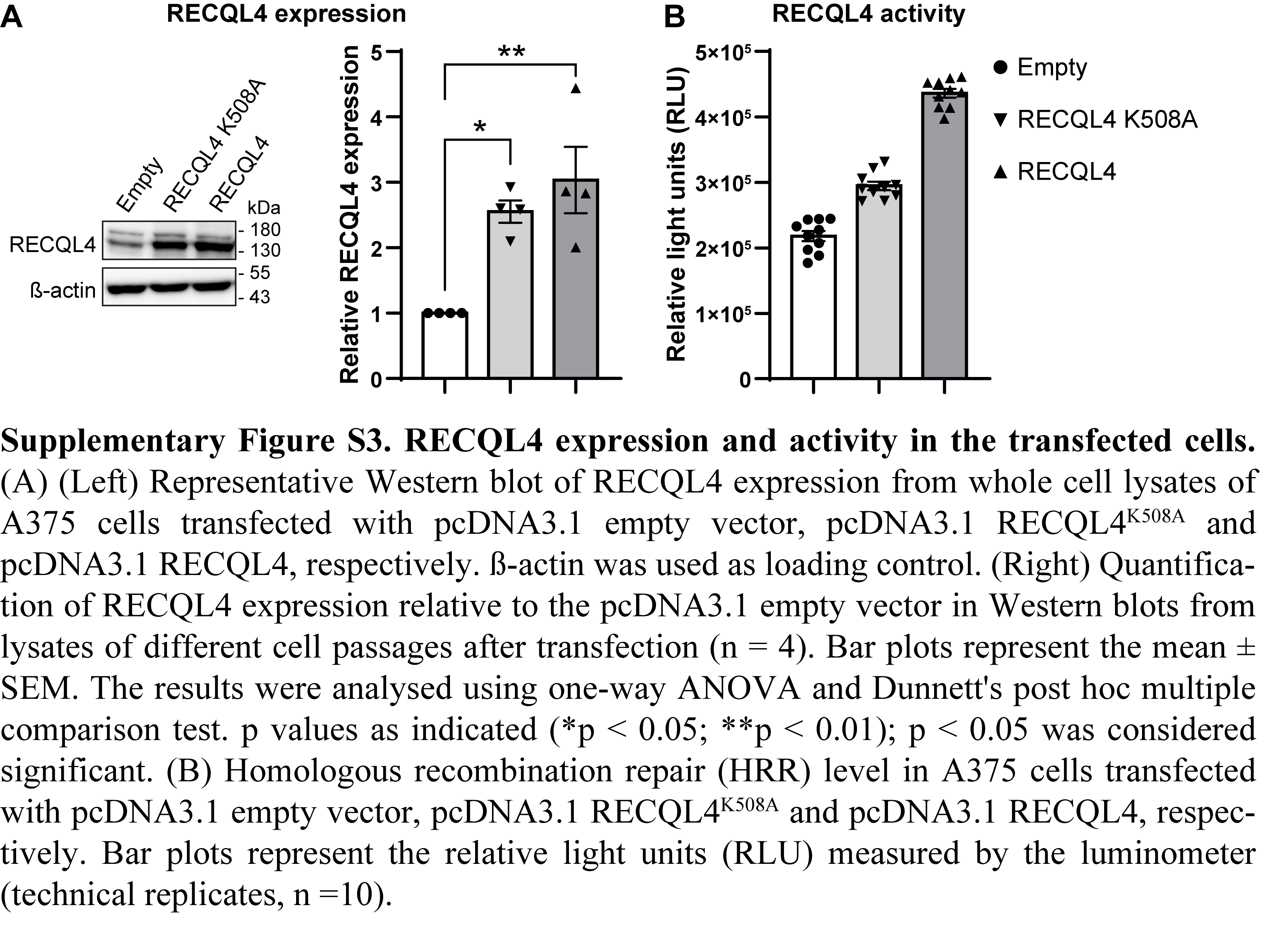

Supplement: Supplementary file 3 — Supporting Information [file CTM2-15-e70094-s004.tif]

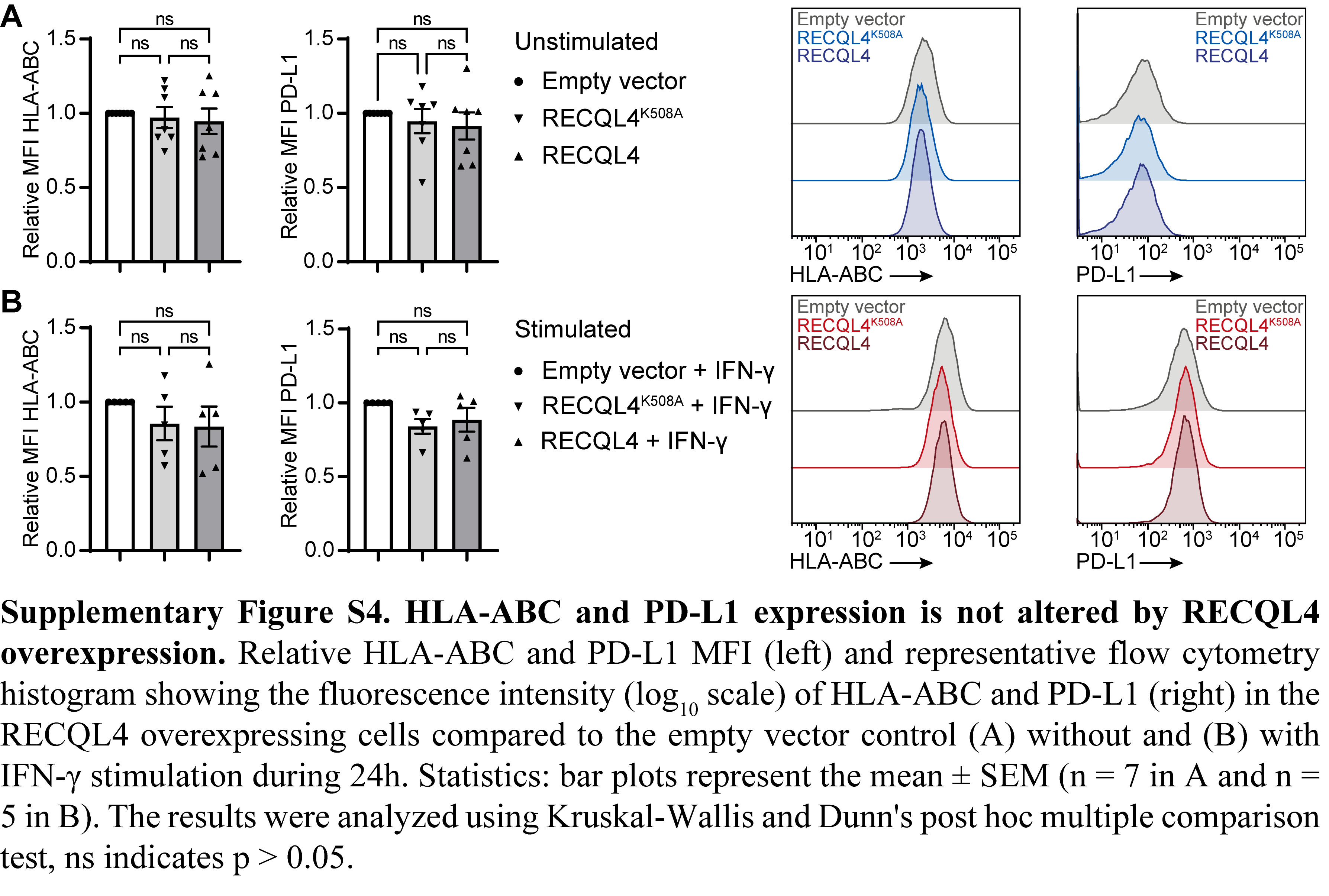

Supplement: Supplementary file 4 — Supporting Information [file CTM2-15-e70094-s001.tif]
